# The Effectiveness of Post-exercise Stretching in Short-Term and Delayed Recovery of Strength, Range of Motion and Delayed Onset Muscle Soreness: A Systematic Review and Meta-Analysis of Randomized Controlled Trials

**DOI:** 10.3389/fphys.2021.677581

**Published:** 2021-05-05

**Authors:** José Afonso, Filipe Manuel Clemente, Fábio Yuzo Nakamura, Pedro Morouço, Hugo Sarmento, Richard A. Inman, Rodrigo Ramirez-Campillo

**Affiliations:** ^1^Centre for Research, Education, Innovation and Intervention in Sport, Faculty of Sport of the University of Porto, Porto, Portugal; ^2^Escola Superior Desporto e Lazer, Instituto Politécnico de Viana do Castelo, Rua Escola Industrial e Comercial de Nun'Álvares, Viana do Castelo, Portugal; ^3^Instituto de Telecomunicações, Delegação da Covilhã, Covilhã, Portugal; ^4^Research Center in Sports Sciences, Health Sciences and Human Development (CIDESD), University Institute of Maia (ISMAI), Maia, Portugal; ^5^Associate Graduate Program in Physical Education Universidade de Pernambuco (UPE)/Universidade Federal da Paraíba (UFPB), João Pessoa, Brazil; ^6^Superior School of Education and Social Sciences, Polytechnic of Leiria, Leiria, Portugal; ^7^Research Unit for Sport and Physical Activity (CIDAF), Faculty of Sport Sciences and Physical Education, University of Coimbra, Coimbra, Portugal; ^8^The Psychology for Positive Development Research Center (CIPD), Universidade Lusíada, Porto, Portugal; ^9^Human Performance Laboratory, Department of Physical Activity Sciences, Universidad de Los Lagos, Osorno, Chile; ^10^Centro de Investigación en Fisiología del Ejercicio, Facultad de Ciencias, Universidad Mayor, Santiago, Chile

**Keywords:** flexibility, post exercise recovery, myalgia, cool-down, delayed onset muscular soreness, stretching, muscle stretching exercises, articular range of motion

## Abstract

**Background:** Post-exercise (i.e., cool-down) stretching is commonly prescribed for improving recovery of strength and range of motion (ROM) and diminishing delayed onset muscular soreness (DOMS) after physical exertion. However, the question remains if post-exercise stretching is better for recovery than other post-exercise modalities.

**Objective:** To provide a systematic review and meta-analysis of supervised randomized-controlled trials (RCTs) on the effects of post-exercise stretching on short-term (≤1 h after exercise) and delayed (e.g., ≥24 h) recovery makers (i.e., DOMS, strength, ROM) in comparison with passive recovery or alternative recovery methods (e.g., low-intensity cycling).

**Methods:** This systematic review followed PRISMA guidelines (PROSPERO CRD42020222091). RCTs published in any language or date were eligible, according to P.I.C.O.S. criteria. Searches were performed in eight databases. Risk of bias was assessed using Cochrane RoB 2. Meta-analyses used the inverse variance random-effects model. GRADE was used to assess the methodological quality of the studies.

**Results:** From 17,050 records retrieved, 11 RCTs were included for qualitative analyses and 10 for meta-analysis (*n* = 229 participants; 17–38 years, mostly males). The exercise protocols varied between studies (e.g., cycling, strength training). Post-exercise stretching included static stretching, passive stretching, and proprioceptive neuromuscular facilitation. Passive recovery (i.e., rest) was used as comparator in eight studies, with additional recovery protocols including low intensity cycling or running, massage, and cold-water immersion. Risk of bias was high in ~70% of the studies. Between-group comparisons showed no effect of post-exercise stretching on strength recovery (ES = −0.08; 95% CI = −0.54–0.39; *p* = 0.750; *I*^2^ = 0.0%; Egger's test *p* = 0.531) when compared to passive recovery. In addition, no effect of post-exercise stretching on 24, 48, or 72-h post-exercise DOMS was noted when compared to passive recovery (ES = −0.09 to −0.24; 95% CI = −0.70–0.28; *p* = 0.187–629; *I*^2^ = 0.0%; Egger's test *p* = 0.165–0.880).

**Conclusion:** There wasn't sufficient statistical evidence to reject the null hypothesis that stretching and passive recovery have equivalent influence on recovery. Data is scarce, heterogeneous, and confidence in cumulative evidence is very low. Future research should address the limitations highlighted in our review, to allow for more informed recommendations. For now, evidence-based recommendations on whether post-exercise stretching should be applied for the purposes of recovery should be avoided, as the (insufficient) data that is available does not support related claims.

**Systematic Review Registration:** PROSPERO, identifier: CRD42020222091.

## Introduction

Exercise sessions typically begin with a warm-up period, followed by the main workout, and end with a cool-down phase, including a progressive reduction of effort and intensity (ACSM, 2018). Stretching is prescribed as an essential component of the cool-down phase by the guidelines of ACSM (2018) and the American Heart Association (2020). The main goals of stretching exercises applied during the cool-down phase (i.e., post-exercise stretching) are to enhance range of motion (ROM) and to reduce stiffness and delayed onset muscle soreness (DOMS) (Sands et al., 2013). There are different post-exercise stretching methods, such as passive static, active static, dynamic, proprioceptive neuromuscular facilitation (PNF), among others (Lima et al., 2019). Despite its wide adoption in exercise protocols, its effectiveness is not well-understood (Van Hooren and Peake, 2018).

Past research has a mixed and often contradicting set of results, with numerous studies indicating post-exercise stretching is not effective for improving recovery. Indeed, in one study with 10 healthy men (Mika et al., 2007), the participants performed three sets of leg extension and flexion at 50% of maximum voluntary contraction (MVC). Post-exercise recovery protocols were used, including light-intensity cycle ergometer and PNF stretching for 5 min. Light-intensity cycle ergometer exercise (10 W at 60 rpm) induced greater short-term recovery (i.e., immediately after the post-exercise protocol) than stretching as measured by MVC, total effort time, motor unit activation and EMG frequency (*p* < 0.05). In another study (Robey et al., 2009), club (8 men, 6 women; age: 20.2 ± 2.2 years) and elite level rowers (4 men, 2 women, age: 18.6 ± 0.8 years) performed a strenuous stair-climb running protocol. Post-exercise recovery protocols were applied at 15-min, 24 and 48 h, including stretching, hot/cold water immersion and passive recovery (i.e., rest). Compared to passive recovery, stretching and hot/cold water immersion induced no recovery effect on leg extension concentric peak torque, 2 km rowing ergometer times, creatine kinase levels, or DOMS, at any time-point. Further, nine physically active men (age, 23 ± 1 years) performed a fatiguing exercise protocol (i.e., 8-min of cycle ergometer at 90% maximum oxygen uptake), followed by a post-exercise stretching protocol (i.e., 10 min) (Cè et al., 2013). After 1 h of performing the stretching protocol, mechanical and physiological assessments (e.g., MVC, EMG amplitude, and lactate kinetics) were similar between the stretching group and the passive recovery group.

Moreover, stretching may be ineffective in relieving perceived muscle pain or in reducing DOMS (Wessel and Wan, 1994; Cheung et al., 2003; Xie et al., 2018). Also, recovery may not simply mean a return to basal values. In other words, to be effective, post-exercise stretching should recover and improve participants function over basal condition (Sands et al., 2013; Van Hooren and Peake, 2018).

Furthermore, potential short-term positive effects of post-exercise stretching on recovery should be balanced with long-term adaptations. For example, Fuchs et al. (2020) recently demonstrated that post-exercise cooling (i.e., cold-water immersion) accelerated acute recovery after training sessions; however, it impaired myofibrillar protein synthesis rates after 2-weeks of training compared to not performing cold-water immersion. In this sense, to comprehensively assess the effectiveness of post-exercise stretching, both short-term and delayed recovery should probably be considered.

In order to bring clarity to conflicting results, systematic reviews and meta-analysis (SRMA) are usually performed as a cornerstone for evidence-based practices (Higgins et al., 2019). Indeed, studies in the field tend to use small samples with reduced statistical power (Abt et al., 2020). In contrast, SRMA provide greater statistical power. In fact, some attempts were performed to synthesize current literature related to post-exercise stretching and recovery. A SRMA of randomized and quasi-randomized studies showed that stretching before or after exercise did not protect from DOMS (Herbert and Gabriel, 2002), and two independent updates reinforced the same conclusions (Henschke and Lin, 2011; Herbert et al., 2011). However, relevant databases such as PubMed and Web of Science were not included in the searches of the aforementioned SRMAs, and potentially relevant search terms such as “mobility” and “post-exercise” or “post-training” were not applied. Likewise, external experts were not consulted after automated searches, as suggested in high-standard protocols (Moher et al., 2009, 2015; Shea et al., 2017). Moreover, nearly a decade has passed since the publication of the aforementioned SRMAs, and a cursory search of articles in Google Scholar from 2011 to present date suggests that several new studies have been done on the topic. An updated SRMA focused solely on post-exercise stretching and limited to randomized controlled trials (RCTs) may provide a more homogeneous and high-quality data set (Hariton and Locascio, 2018), while an expanded set of relevant databases and search terms may provide a more representative sample of existing studies.

Therefore, our goal was to review supervised RCTs on the effects of post-exercise stretching on recovery makers (i.e., DOMS, strength, ROM), in comparison with passive recovery or alternative recovery methods (e.g., low-intensity cycling). Short-term (≤1 h after exercise) and delayed recovery (24, 48, and 72 h) markers were considered.

## Methods

### Protocol and Registration

This systematic review followed the Preferred Reporting Items for Systematic Reviews and Meta-Analyses (PRISMA) guidelines (Moher et al., 2009, 2015), the Cochrane Collaboration guidelines for evaluation of risk of bias (RoB) in randomized studies (Sterne et al., 2019), and the AMSTAR 2 recommendations (Shea et al., 2017). Quality of studies was assessed using the Grading of Recommendations Assessment, Development, and Evaluation (GRADE) (Guyatt et al., 2011). The review methods were established before initiating the research, and protocol registration preceded the search. Protocol was published in PROSPERO with the reference CRD42020222091.

### Eligibility Criteria

Studies were eligible if consisting of original research or replication studies published in peer-reviewed journals, with full-text not limited to any particular language or publication date. Beyond English language, the authors also have a deep understanding of Portuguese and Spanish, as well as a good understanding of French and Italian. If studies were written in different languages, professional translators were hired. Based on scope, P.I.C.O.S. and timeframe for follow-up, Table 1 presents the inclusion and exclusion criteria. The limitation to RCTs was decided because randomization reduces the RoB and balances participants distribution between groups (Hariton and Locascio, 2018). Indeed, RCTs are the gold standard for evidence-based practices (Spieth et al., 2016). Supervision was considered if explicit information was available stating that at least one qualified professional oriented the post-exercise protocol. No studies were excluded on the basis of RoB as assessed through RoB 2 (Sterne et al., 2019).

**Table 1:** Inclusion and exclusion criteria based on scope, PICOS and timeframe for follow-up.

**Rule**	**Inclusion criteria**	**Exclusion criteria**
Study type	Original research or replication studies published in peer-reviewed journals. No limitations imposed regarding language or publication date.	Conference abstracts, books and book chapters, editorials, letters to the editor, feasibility and pilot studies, trial registrations, reviews, essays, or original research in non-peer-reviewed journals.
Participants	Participants of any age, sex, health, and training status.	Non-human animals (e.g., rats).
Interventions	Stretching (e.g., static passive, static active, dynamic, PNF, other) immediately after any type of exercise session (e.g., strength training, endurance, multimodal, sports). No co-interventions.	Stretching as the training intervention *per se*. Pre-exercise stretching (e.g., warm-up). Post-exercise multimodal interventions (e.g., stretching combined with low-intensity cycling). Post-exercise stretching with co-interventions (e.g., massage).
Comparators	Passive recovery (i.e., rest) or alternative recovery protocols (e.g., low-intensity aerobic activities, massage).	Absence of comparators. Multimodal comparators that include stretching.
Outcomes	*Primary outcomes* Effects on short-term post-exercise recovery (≤1 h post-exercise): strength, DOMS, ROM. Effects on delayed post-exercise recovery (24, 48, 72 h)^*^: strength, DOMS, ROM. *Secondary outcomes* Biochemical markers of muscle damage; muscle and tendon stiffness; adverse effects from the post-exercise interventions.	No outcomes related to strength and/or ROM for short-term recovery. AND No outcomes related to DOMS, strength and/or ROM for delayed recovery.
Study design	Supervised RCTs (parallel or cross-over) (Elbourne et al., 2002; Spieth et al., 2016).	Non-randomized studies. Non-supervised intervention and/or comparators. Case reports, case series, observational studies (e.g., case-control and cohort studies).
Timeframe for follow-up	Maximum 72 h post-intervention, based on the existing literature (Van Hooren and Peake, 2018).	No study will be excluded if presenting values >72 h, but these will not be considered for analysis.

*DOMS, delayed onset muscular soreness; PNF, proprioceptive neuromuscular facilitation; RCT, randomized controlled trial; ROM, range of motion*.

* *If an additional exercise bout or an active recovery protocol is included between the initial session and the delayed markers (e.g., application of a second exercise bout at 48 h while providing data regarding recovery from the initial bout at 72 h), then only values until that second bout (i.e., 48 h) will be considered*.

### Information Sources

Search was programmed to start on January 1st, 2021, but since protocol approval occurred earlier (December 2nd, 2020), we conducted the automated searches on December 23 and 24, 2020, with search results being exported to EndNote X9 for Mac (v.9.3.3., Clarivate Analytics). The following electronic databases were searched: Cochrane Library (including CENTRAL), EBSCO (all available databases), PEDro, PubMed, Scielo, Scopus, SPORTDiscus (all databases), and Web of Science (all databases/collections). Search protocol used Boolean operators and required the title, abstract, or keywords had to include (“stretch^*^” OR “flex^*^”* OR “mobility” OR “range of motion”)* AND (“post-exerci^*^” OR “post-workout” OR “post-exertion” OR “post-train^*^” OR “after exerci^*^” OR “after workout” OR “after exertion” OR “after training” OR “recover^*^”* OR “warm-down” OR “cool-down”)* AND “*random*.^*^” Similar terms or synonyms were used to guarantee a more inclusive initial search and avoid an excessively narrow scope of analyzed studies. Searches were updated on February 16, 2021, for inclusion of records with date of entry from December 25, 2020, onwards. Where date of entry was not a feature (e.g., EBSCO, Scielo, Scopus, SPORTDiscus, Web of Science), publication date was limited to 2021, since the year 2000 would be practically all covered until the search was completed.

A manual search was conducted within the reference list of the records included in the sample after full text analysis, to retrieve potentially relevant studies that had not emerged in the initial search. After completion of this stage, the list of studies, as well as inclusion and exclusion criteria were sent to eight independent experts in the field, to check if they were aware of additional papers. The experts were university professors with a Ph.D. and with peer-reviewed publications within the scope of our SRMA. Search strategy was not provided, to avoid biasing the experts' search. After the final list of studies was completed, all the databases were again consulted to retrieve errata, corrigenda/corrections, or retractions of the included studies, as some may have been found to be fraudulent or retracted (Higgins et al., 2019).

### Study Selection

The screening process started on January 4, 2021 for the first wave of searches. The screening process for the updated searches started on February 17, 2021. JA and FMC conducted the initial search, screening of titles and abstracts and analysis of full texts independently. HS and PM later reviewed the entire process. Thirdly, a step-by-step comparison of the whole process was conducted, and any disagreements motivated a new analysis of the records in question. Discussion regarding manuscripts suitability was performed with all the involved authors in the study selection process, until consensus was achieved. The same process was then used to analyze the reference lists of the included studies to verify if additional relevant studies were available. External experts were contacted to provide additional suggestions of relevant studies based on inclusion criteria and on our preliminary list. JA and FMC independently verified the list to decide on inclusion of the suggested studies. HS and PM then reviewed this process. The same process was applied to search for errata of the included studies.

### Data Extraction

All extracted data were defined *a priori*, to avoid biased analyses (Spieth et al., 2016). Study characteristics: (i) sample size and features (e.g., age, sex, health, training status, country, continent; single or multicenter study); (ii) length and characteristics of the interventions and comparators (e.g., weekly frequency, type/modality of stretching and comparators, volume, intensity, duration, supervision ratio, qualification of supervisors, description of co-interventions); (iii) adherence rates to training (i.e., attendance percentage); (iv) funding sources and potential conflicts of interest. Data specific to cross-over studies (Elbourne et al., 2002; Spieth et al., 2016): (i) length of wash-in and wash-out periods; (ii) carryover effects, if there were any.

*Primary outcomes for short-term recovery* (≤1-h post-intervention): strength levels (e.g., maximum voluntary contraction) and joint ROM immediately or until 1 h after exertion. *Primary outcomes for delayed recovery*: DOMS, strength levels, and joint ROM at 24, 48, and 72 h, which are considered theoretically relevant (Van Hooren and Peake, 2018) and are commonly assessed periods on studies investigating this subject matter (Bonfim et al., 2010; Torres et al., 2013).

*Secondary outcomes*: Biochemical markers (e.g., plasma creatine kinase; blood lactate concentration); muscle and tendon stiffness; adverse effects during the post-exercise interventions (type, intensity or severity, time points). The timings described in the previous paragraph were considered for secondary outcomes as well.

Outcomes were only considered for analysis in case there was no additional exercise bout between the initial session and the delayed recovery timeframe. For all primary and secondary outcomes, description of measurement tools and metrics was included (Higgins et al., 2019) and both significant and non-significant results were considered (Spieth et al., 2016). Furthermore, parallel and cross-over trials were combined as long as the latter did not have significant carryover effects (Elbourne et al., 2002). JA and FMC completed initial data extraction independently. HS and PM later reviewed the entire process and consensus had to be achieved. The data required for meta-analysis was fulfilled by JA and FMC and then reviewed by HS and PM. RRC provided a final verification of the quality of data inserted into the table.

### Risk of Bias in Individual Studies

Bias refers to systematic errors that can threaten the internal validity of an RCT (Spieth et al., 2016). RoB was assessed using the revised Cochrane risk-of-bias tool for randomized trials (RoB 2) (Sterne et al., 2019), which consists of five dimensions, i.e., bias arising due to: (i) the randomization process; (ii) deviations from intended interventions; (iii) missing outcome data; (iv) measurement of the outcome; and (v) selection of the reported result. JA and FMC independently assessed RoB for all studies. After the first assessment, tables were compared and disagreements were discussed, with a subsequent re-analysis of the situation. Finally, HS and PM reviewed the assessments to ensure the quality of the evaluations. For assessing RoB in parallel trials, the Excel tool ROB2_IRPG_beta_v7 (Cochrane) was used. For crossover trials, the Excel tool ROB2.0_IRCX_beta (MRC | Hubs for Trials Methodology Research) was planned to be used. However, this tool is outdated. Following the most up-to-date Cochrane guidelines for applying RoB 2 to individual cross-over trials (Higgins et al., 2020), the five domains can be assessed following the structure of parallel trials. However, an extra dimension (Domain S) is added. Therefore, we used the ROB2_IRPG_beta_v7, with manual addition of Domain S.

### Summary Measures

It is possible to use two studies in a meta-analysis (Valentine et al., 2010), but we chose to establish a minimum of three studies (Moran et al., 2018; García-Hermoso et al., 2019; Skrede et al., 2019) to avoid small sample sizes (Abt et al., 2020; Lohse et al., 2020). Pre- and post-intervention means and standard deviations (SDs) were converted to Hedge's *g* effect size (ES) (García-Hermoso et al., 2019; Skrede et al., 2019). In case the study instead provides 95% confidence intervals (CIs) or standard errors of mean (SEM), means and standard deviations were obtained from 95% CI or SEM, using Cochrane's RevMan Calculator for Microsoft Excel (Drahota and Beller, 2020). In case data for primary outcomes was presented only in graphical form, a validated software (*r* = 0.99, *p* < 0.001), WebPlotDigitizer, version 4.4 (Rohatgi, 2020) was used to extract data, with all values rounded to two decimal places. In these cases, the main author extracted data from the graphs, and an outside researcher, not involved in this work (see section Acknowledgments), performed an independent data extraction. Reliability was calculated through Cronbach's Alpha, using SPSS Statistics version 27 for Mac (IBM).

The inverse variance random-effects model for meta-analyses was used because it allocates a proportionate weight to trials based on the size of their individual standard errors (Deeks et al., 2008) and enables analysis while accounting for heterogeneity across studies (Kontopantelis et al., 2013). The ESs were presented alongside 95% CIs and interpreted using the following thresholds (Hopkins et al., 2009): <0.2, trivial; 0.2–0.6, small; >0.6–1.2, moderate; >1.2–2.0, large; >2.0–4.0, very large; >4.0, extremely large. Heterogeneity was assessed using the *I*^2^ statistic, with values of <25, 25–75, and >75% considered to represent low, moderate, and high levels of heterogeneity, respectively (Higgins and Thompson, 2002). Publication bias was explored using the extended Egger's test (Egger et al., 1997). To adjust for publication bias, a sensitivity analysis was conducted using the trim and fill (Duval and Tweedie, 2000), with L0 as the default estimator for the number of missing studies (Shi and Lin, 2019). Analyses were performed in the Comprehensive Meta-Analysis program (version 2; Biostat, Englewood, NJ, USA). Statistical significance was set at *p* ≤ 0.05.

### Moderator Analyses

These analyses were planned but could not be performed. Details on planned moderator analysis can be found in the Supplementary Materials.

### Confidence in Cumulative Evidence

For RCTs, GRADE starts assuming high quality, which can be downgraded according to five dimensions (Zhang et al., 2019). In addition to RoB, inconsistency (heterogeneity) and publication bias, which have already been addressed, indirectness and imprecision (using 95% CIs) were assessed independently by JA and FMC and verified by HS. These authors also estimated the overall quality and confidence in cumulative evidence.

## Results

### Study Selection

Initial search retrieved 16,851 results [Cochrane Library: 13 reviews and 621 trials; EBSCO: 1,704; PEDro: 21; PubMed: 2,421; Scielo: 12; Scopus: 5,253; SPORTDiscus: 734; Web of Science (all collections): 6,072]. Automated removal (EndNote function) of 6,635 duplicates resulted in 10,216 records. Manual removal of additional 2,333 duplicates resulted in 7,882 records to be screened. The first stage of screening titles and abstracts was based on study type (first inclusion criteria) and resulted in the exclusion of 2,101 records. The second stage of screening started with 5,781 records and 5,481 studies that were clearly out of scope (e.g., exercise-related studies not addressing the theme of our work, non-exercise related studies) were removed. Finally, starting with 300 records, the third stage of screening applied the PICOS criteria, and further excluded 278 studies. In these three stage-screening processes, exclusion criteria were defined hierarchically, i.e., if a paper had several reasons for exclusion, its exclusion would be based on the first criteria it failed to fit. Finally, two records had untraceable full texts, with discontinued links, disappearance from databases from where they were retrieved, and even not emerging in searches within the journals where they were supposedly published.

The updated searches retrieved 199 new records [Cochrane Library: 1 review and 8 trials; EBSCO: 49; PEDro: 0; PubMed: 53; Scielo: 3; Scopus: 25; SPORTDiscus: 7; Web of Science (all collections): 53]. Removal of duplicates results in 121 records, of which 14 were excluded due not fitting study type, 60 being non-related to exercise, 40 being related to exercise but out of scope, and six did not comply with PICOS criteria. More in-depth information concerning the screening can be found in Supplementary Table 1. Therefore, 21 records were considered eligible for analysis of the full text (20 in the initial searches and one in the updated searches). While most were written in English, one was in Portuguese (Bonfim et al., 2010), one in Greek (Kokkinidis et al., 1998), and three in Korean (
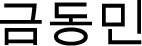
 et al., 2010; Oh, 2013; Kang and Park, 2018). A translator was hired for the Korean studies, and another for the Greek study.

At this stage, 12 records were excluded, with reasons. The study by Apostolopoulos et al. (2018) was excluded because the interventions were not supervised. However, they have interesting results that we will explore briefly here. Since they applied stretching for three consecutive days after the eccentric exercise protocol, only results at 24 h were considered. The authors used a 90% CIs (and not the more common 95% CIs) to compare low-intensity and high-intensity stretching to a control group using passive rest. Despite the authors' claims, all 90% CIs passed through zero, and no differences were observed at 24 h between the stretching groups and the controls for DOMS, eccentric and isometric peak torques of knee extensors, creatine kinase (U/L), and high-sensitivity C-reactive protein. The study of Boobphachart et al. (2017) was excluded because the stretching intervention was performed three times per day and, furthermore, was unsupervised.

The study of Cha and Kim (2015) was excluded because both groups included some form of stretching, therefore inhibiting the comparison of stretching with alternative protocols and failing our PICOS criteria. The study of Duffield et al. (2014) was excluded because both the training interventions and the protocols were applied twice a day. Furthermore, one of the protocols included not only immediate measures (15-min cold-water immersion), but also ongoing measures such as 3 h of wearing full-body compression garments, plus abiding by sleep-hygiene recommendations in that night. The study of Gulick et al. (1996) was excluded because randomization was compromised. The authors created seven groups with 10 participants each. When a participant would quit, they would simply recruit a new participant to the group, therefore compromising both randomization and baseline values for each group. In addition, no details were provided concerning how these new subjects changed the values for each variable.

The study of Kang and Park (2018) was excluded because the exercise intervention lasted 20 min, while the post-exercise stretching protocol consisted of 5 min of so-called preparation exercises, followed by 30 min of stretching, followed by 5 min of so-called clean-up exercises. Therefore, not only did post-exercise recovery last 200% more than the exercise intervention (thereby, being akin to a stretching intervention *per se* and failing our inclusion criteria), but also the recovery intervention was not exclusively reliant on stretching (again, falling our inclusion criteria). The study of McGlynn et al. (1979) was excluded because stretching was applied immediately post-exercise, but also repeated at 6, 25, 30, 49, and 54 h post-exercise. Therefore, even the 24 h assessments could not be attributed to stretching performed immediately following an exercise bout. Incidentally, the authors reported that both the stretching and biofeedback groups observed a reduction in EMG muscle activity on the biceps brachii in comparison with a passive control group, but they had no effect on perceived pain.

The study of Oh (2013) was excluded because the cool-down protocols were not stretching-based. The study of Pooley et al. (2020) was a cross-over study that was excluded because randomization was compromised: while after “home” fixtures, the participants were randomized to cold-water immersion or cycle ergometer, in “away” fixtures stretching was always prescribed. The study of Robey et al. (2009) was excluded because the authors detail, in the manuscript, that the crossover was only semi randomized, and therefore does not meet our inclusion criteria. In any case, the main characteristics and results from this study have been addressed in the introduction, which was written prior to our searches. The study of Xanthos et al. (2013) was excluded because the so-called traditional recovery group was multimodal. The study of 
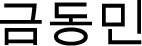
 et al. (2010) was excluded because the cool-down protocol was multimodal.

Therefore, nine studies fulfilled all inclusion criteria (Kokkinidis et al., 1998; Mika et al., 2007; Bonfim et al., 2010; Cè et al., 2013; Torres et al., 2013; McGrath et al., 2014; Muanjai and Namsawang, 2015; Cooke et al., 2018; César et al., 2021). As per protocol, in studies where the recovery methods were applied in multiple sessions (e.g., stretching after exercise and repeated at 24 and 48 h), only data before the second application was considered. To illustrate, in the studies of Cooke et al. (2018) and Kokkinidis et al. (1998), only the results at 24 h post-exercise were considered. Since a new recovery session was applied at 24 h, the results at 48 h and longer were not considered in the meta-analysis since results might not be attributable to the immediate post-exercise stretching protocol. In addition, and following protocol, multimodal recovery groups also including stretching were excluded from analysis (e.g., the group combining stretching followed by cold water immersion in the study of Muanjai and Namsawang, 2015). In the study of Torres et al. (2013), two groups were considered: the group performing eccentric exercise, and the group performing eccentric exercise followed by a single bout of stretching. The group that only performed stretching and the group that performed eccentric exercise followed by repeated bouts of stretching in the following days were excluded as they did not conform to our inclusion criteria.

**Figure 1:**
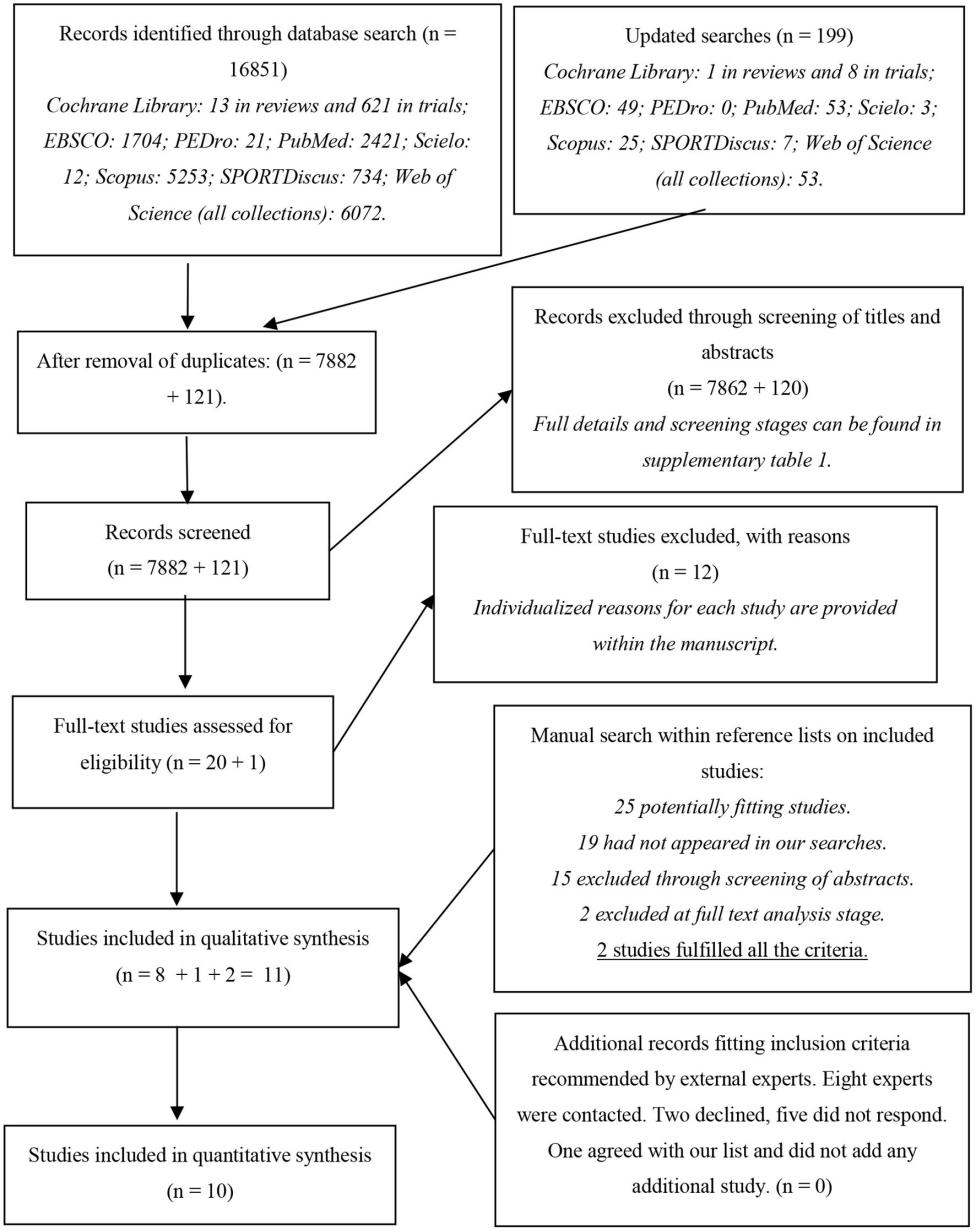
Flowchart describing the study selection process.

A manual search within the reference lists of included studies revealed 26 potentially fitting titles (including updated searches). Of these, two had already been included in our final sample, and five had been excluded during the process. Nineteen studies had not appeared in our searches; screening of their abstracts resulted in the exclusion of five based on study type (e.g., abstract, review), and 10 based on failure to fulfill PICOS criteria. Of the four studies that required full text analysis, two fulfilled all PICOS criteria and were therefore added to our sample (Torres et al., 2005; West et al., 2014). In relation to Torres et al. (2005), and following the rules applied to Torres et al. (2013), only the two groups meeting the criteria were considered for analysis. Subsequently, eight experts were invited to contribute with additional relevant studies. Two experts declined the invitation due to lack of time, while five experts did not respond. One expert responded that our list was thorough and did not make any additional recommendation. Finally, errata, corrigenda, corrections, and retractions were searched for the included studies, but none was found. Therefore, 11 studies were included for qualitative analysis (*n* = 289), of which 10 could integrate quantitative analysis (*n* = 280, *n* = 229 after exclusion of groups that did not fulfill PICOS criteria). The process is summarized in the PRISMA flow diagram (Figure 1).

### Study Characteristics

Study characteristics are provided in Table 2. Three studies used a cross-over design (Mika et al., 2007; Cè et al., 2013; West et al., 2014), while the remaining used a parallel design. Sample size ranged from 9 (Cè et al., 2013) to 57 (McGrath et al., 2014), with ages ranging from 17 to 38 years-old, i.e., all studies were performed with adults or near-adulthood (i.e., the usual legal age of 18 years old). The studies of Bonfim et al. (2010) and McGrath et al. (2014) had a mixed sample of men and women. The remaining studies only used male participants. All participants were healthy, but varied considerably in terms of training status: described as sedentary or untrained in four studies (Kokkinidis et al., 1998; Torres et al., 2005, 2013; Bonfim et al., 2010), “physically active,” “recreationally active,” or “not involved in intense physical conditioning” in five studies (Mika et al., 2007; Cè et al., 2013; McGrath et al., 2014; Muanjai and Namsawang, 2015; Cooke et al., 2018), one study assessed the effects in aerobically trained, recreational cyclists (West et al., 2014), and only one study assessed athletes (César et al., 2021). Geographically, five studies were performed in Europe (Kokkinidis et al., 1998; Torres et al., 2005, 2013; Mika et al., 2007; Cè et al., 2013), three in North America (McGrath et al., 2014; West et al., 2014; Cooke et al., 2018), two in South America (Bonfim et al., 2010; César et al., 2021) and one in Asia (Muanjai and Namsawang, 2015).

**Table 2:** Study characteristics.

**Authors (year)/country**	**Trial design**	**Sample**	**Intervention**	**Length**	**Intervention (stretching group)**	**Comparators**	**Funding and potential conflicts of interest**
Bonfim et al. (2010) Brazil	P	20 M/F healthy sedentary [17–30 years]	After 5 × 20 rep (with 30-s rest) of plantar/dorsiflexion, while standing and hands providing anchoring, EG (*n* = 10) performed active static stretching for ± 3 min.	Single application	*n* = 10. Active static stretching, 3 × 30-s per limb, per set. Until subjects perceived a slight feeling of stretching of the triceps surae, without generating discomfort.	*n* = 10. Passive recovery (rest).	No mention to funding. Explicit statement reporting no potential conflicts of interest.
Cè et al. (2013) Italy	CO	9 male healthy active (23 ± 1.0 years)	10-min warm-up followed by 8-min of cycling in ergometer at 90% VO_2_max. Then, 10-min of passive static stretching.	Unclear, but estimated 2–3 sessions per week, during ~3 weeks	*n* = 9. Passive static stretching, 5 × 30-s per set, with 30-s rest between sets. Stretching to the point of discomfort.	*n* = 9. In a CO manner, for 10 min: superficial massage, deep massage, passive recovery (rest), low-intensity cycling (50% VO_2_max).	No mention to funding. Explicit statement reporting no potential conflicts of interest.
César et al. (2021) Brazil	P	21 male healthy jiu-jitsu fighters (27.0 ± 5.9 years)	After 10-min of fight, EG (*n* = 7) performed passive static stretching for 10-min.	1 week	*n* = 7. Passive static stretching, 9 × 30-s per limb, per set. Until the greatest discomfort was reported by the participants.	*n* = 7 + 7. CWI (3 × 3 min at 10°C) and passive recovery (rest).	No mention to funding. No mention to potential conflicts of interest.
Cooke et al. (2018) USA	P	25 male healthy active (22.2 ± 3.5 years)	After 10-min warm-up and 45-min downhill running exercise, EG (*n* = 9) performed active static stretching for 30-min.	4 visits to the lab	*n* = 9. Active static stretching, 3 × 30-s per set. Stretching to the point of mild discomfort, but not pain.	*n* = 8 + 8. 30-min jogging at 60% VO_2_peak and 0° incline. Treadmill + anti-gravity treadmill (at 75% bodyweight).	Partially funded by authors' lab, and by the company manufacturing the anti-gravity treadmill. Conflicts of interest not mentioned by the authors, but with financial support by the treadmill company.
Kokkinidis et al. (1998) Greece	P	12 male healthy sedentary [18–27 years]	After 6 × 10 rep (with 3-min rest) of leg curl focusing on the eccentric part of the movement, EG (*n* = 4) performed active static stretching for ±12-min.	3 days	*n* = 4. Active static stretching, 10 × 30-s per set, with 10-s rest between sets. Until subjects felt a painless sensation of stretching.	*n* = 4 + 4. Cryotherapy (cold compresses for 20 min) + passive recovery (rest).	No mention to funding. No mention to potential conflicts of interest.
McGrath et al. (2014) USA	P	29 M and 28 F healthy active [“Substantial majority” between 18–25 years]	After 5–10 min warm-up, 3 × 8–12 rep leg curl with an 8–12 RM load, focusing on the eccentric phase, EG (*n* = 20) performed passive static stretching for ±1-min.	Single application	*n* = 20 (14 male, 6 female). Passive static stretching, 2 × 10-s per limb, per set, with 4-s rest between sets. Until subjects felt a maximal stretch of the hamstrings.	*n* = 19 (9 male, 10 female) + 18 (5 male, 13 female). PNF (5-s each phase) + passive recovery (rest).	No mention to funding. No mention to potential conflicts of interest.
(Mika et al., 2007) Poland	CO	10 male healthy active [24–38 years]	3 sets (30-s rest) of dynamic leg extension and flexion (20–110°) at 50% MVC. Subjects had to perform as many repetitions as possible, stopping only when full ROM was no longer achieved. Following, 5-min of PNF stretching was applied.	1 session per week, for 5 weeks	*n* = 10. PNF stretching of unclear duration: 5-s performing isometrics, but unknown time during each passive stretching phase. Passive stretch to the point of onset of resistance, followed by isometric contraction, followed by relaxation and new passive stretch.	*n* = 10 + 10. Cycle ergometer (10 W, 60 rpm, 5-min) + passive recovery.	No mention to funding. No mention to potential conflicts of interest.
Muanjai and Namsawang (2015) Thailand	P	27^a^ male healthy active (20.9 ± 1.1 years)	After plyometric training (3 sets of single leg bound, 6 sets of 30 m double leg bounds, 6 sets of 10 m tuck jumps and 5 sets of 10 drop jumps on a 60 cm box; maximum 10-s rest between each jump and 2-min rest between each set), EG (*n* = 13) performed passive static stretching for 20-min.	Single application	*n* = 13. Passive static stretching, 2 × (5 × 30-s, with 5-s rest) per limb, with 1-min rest between sets. Until a sensation of stretch or resistance against the movement was felt; if this was not achieved, hip extension was added.	*n* = 14. 20-min CWI at 15 ± 1°C. [Stretching + CWI group excluded following PICOS criteria.]	Grant attributed by the National Research Council of Thailand. No mention to potential conflicts of interest.
Torres et al. (2005) Portugal	P	17^b^ male healthy sedentary (21.2 ± 2.2 years)	After eccentric contractions for knee extensors, performed in an isokinetic dynamometer (2 sets of eccentric contractions until fatigue, 30-s rest in-between, at 80% maximum peak torque and 60°/s; range of motion fixed between 20° and 90° of knee flexion), EG (*n* = 9) performed passive static stretching for 6.5-min.	Single application	*n* = 9. Passive static stretching, 10 × 30-s per set, with 10-s rest between sets. Until resistance and/or discomfort were felt; if it was not felt, hip extension was added to knee flexion.	*n* = 8. Passive recovery (rest).	No mention to funding. No mention to potential conflicts of interest.
Torres et al. (2013) Portugal	P	28^c^ male healthy untrained (21.4 ± 1.9 years)	After eccentric contractions for knee extensors, performed in an isokinetic dynamometer (2 sets of eccentric contractions until fatigue, 30-s rest in-between, at 80% maximum peak torque and 60°/s; range of motion fixed between 20° and 90° of knee flexion), EG (*n* = 14) performed passive static stretching for 6.5-min.	Single application	*n* = 14. Passive static stretching, 10 × 30-s per set, with 10-s rest between sets. Until resistance and/or discomfort were felt; if it was not felt, hip extension was added to knee flexion.	*n* = 14. Passive recovery (rest).	Grant attributed by the Foundation for Science and Technology, Portugal. No mention to potential conflicts of interest.
West et al. (2014) USA	CO	12 male healthy trained (21.3 ± 2.3 years)	29-km stationary cycling time trial, followed by 30-min of active static stretching.	1 session every 2 weeks, during ~45 days	*n* = 12. Active static stretching, 3 × 30-s per set. Stretches held to the point of mild discomfort, but not pain.	*n* = 12 + 12. 30-min running in antigravity treadmill (40% VO_2_peak, 75% bodyweight) + cycle ergometer (40% VO_2_peak).	Partially funded by authors' lab, and by the company manufacturing the anti-gravity treadmill. Conflicts of interest not mentioned by the authors, but with financial support by the treadmill company.

*P, Parallel; CO, Crossover; M/F, Male and Female; CWI, Cold-water immersion; EG, Experimental Group; HG, Handgrip; MVC, Maximum voluntary contraction; PNF, Proprioceptive neuromuscular facilitation; ROM, range of motion*.

a *After excluding the subjects of the multimodal recovery group, because it also included stretching and therefore had to be excluded due to PICOS*;

b *After exclusion of Group 1, since stretching was the intervention per se, and not a post-exercise application*;

c *After exclusion of Group 1, since stretching was the intervention per se, and not a post-exercise application, and after exclusion of group 4, which had multiple application/bouts of the recovery intervention*.

The studies purposefully applied soreness-inducing exercise protocols for the upper limbs (César et al., 2021) or lower limbs (all other articles), using diverse means such as cycling (Cè et al., 2013; West et al., 2014), running-based activities (Cooke et al., 2018), plyometrics (Muanjai and Namsawang, 2015), simulated jiu-jitsu fights (César et al., 2021), and more commonly, some form of strength training, usually with an emphasis on the eccentric component (Kokkinidis et al., 1998; Torres et al., 2005, 2013; Mika et al., 2007; Bonfim et al., 2010; McGrath et al., 2014). Familiarization with the soreness-inducing protocols was described in three studies (Mika et al., 2007; Cè et al., 2013; Cooke et al., 2018; César et al., 2021), stated but not described in one (Muanjai and Namsawang, 2015), and not performed or unreported in six (Kokkinidis et al., 1998; Torres et al., 2005, 2013; Bonfim et al., 2010; McGrath et al., 2014; West et al., 2014). In most studies, the duration of the soreness-inducing protocol was unclear (Kokkinidis et al., 1998; Torres et al., 2005, 2013; Mika et al., 2007; Bonfim et al., 2010; McGrath et al., 2014; West et al., 2014; Muanjai and Namsawang, 2015), but unlikely to have surpassed 30 min, considering the descriptions provided. In the remaining studies, soreness-inducing protocols lasted between 10 min (César et al., 2021) and 55 min (Cooke et al., 2018), including warm-up when applied. The only study to report a co-intervention stated that a nutritional bar was provided pre-fatiguing exercise (West et al., 2014).

All studies had at least one group performing post-exercise stretching as an attempt to mitigate the negative effects of the soreness-inducing protocols. Active static stretching was used in four studies (Kokkinidis et al., 1998; Bonfim et al., 2010; West et al., 2014; Cooke et al., 2018), passive stretching in six (Torres et al., 2005, 2013; Cè et al., 2013; McGrath et al., 2014; Muanjai and Namsawang, 2015; César et al., 2021), and PNF in two (Mika et al., 2007; McGrath et al., 2014). McGrath et al. (2014) used both passive static stretching and PNF. No study used dynamic stretching. Almost all the post-exercise stretching protocols targeted the lower limbs, with one study targeting the upper limbs (César et al., 2021), and lasted between ~1 min (McGrath et al., 2014) and 30 min (West et al., 2014; Cooke et al., 2018). Intensity of stretching was measured using only subjective feelings during the exercise, ranging from “subjects perceiving a slight feeling of stretching (…), without generating discomfort” (Bonfim et al., 2010) to “until subjects felt a *maximal* stretch of the hamstrings” (McGrath et al., 2014) or “until the greatest discomfort was reported by the participants” (César et al., 2021).

The comparator post-exercise interventions were also varied across studies, with some studies having more than one comparator group. Passive recovery (i.e., rest) was used as comparator in eight studies (Kokkinidis et al., 1998; Torres et al., 2005, 2013; Mika et al., 2007; Bonfim et al., 2010; Cè et al., 2013; McGrath et al., 2014; César et al., 2021). Additional recovery protocols included low-intensity cycling (Mika et al., 2007; Cè et al., 2013; West et al., 2014) or running/jogging (West et al., 2014; Cooke et al., 2018), superficial and deep massage (Cè et al., 2013), cryotherapy and/or cold-water immersion (Kokkinidis et al., 1998; Muanjai and Namsawang, 2015; César et al., 2021).

One study explicitly stated that there were no adverse effects to report (Muanjai and Namsawang, 2015), while the other studies made no mention to it. We further highlight that two studies had potentially relevant conflicts of interest, as the company manufacturing the anti-gravity treadmill provided financing for the research (West et al., 2014; Cooke et al., 2018).

### Risk of Bias Within Studies

Cochrane's RoB 2 tool evaluates RoB in five different dimensions (Sterne et al., 2019), the second of which subdivided into two parts. Here, an intention-to-treat analysis was considered. In terms of outcomes, RoB was only assessed for the primary outcomes (i.e., strength, ROM, and DOMS). None of the included studies had a pre-registered protocol. However, one had a specific reference to a grant (Torres et al., 2013), and another to an approval number by an Ethics Committee (Bonfim et al., 2010). In both cases, a pre-study protocol had to exist, and so we have contacted the authors. The corresponding author of Bonfim et al. (2010) provided the trial protocol, which also contained a statistical analysis plan. The main author of Torres et al. (2013), which was the recipient of the grant, was contacted, but unfortunately did not have the original project, which is comprehensible given the timeline. Since some studies had more than one outcome, assessments for domains 4 and 5 could have multiple assessments for each study. The complete assessments (i.e., one assessment per outcome per study) can be found in Supplementary Table 2. Table 3 presents the worst-case scenario for each study, i.e., considering the outcome for which the risk of bias was higher.

**Table 3:** Risk of bias in individual studies (worst-case scenario).

**References**	**D1**	**D2**	**D3**	**D4**	**D5**	**DS**	**Overall**
Bonfim et al. (2010)	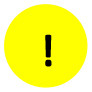	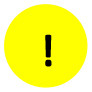	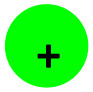	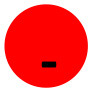	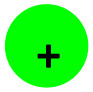	N/A	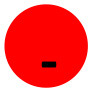
Cè et al. (2013)	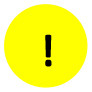	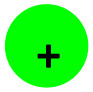	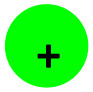	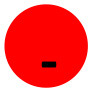	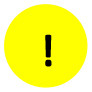	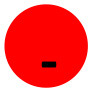	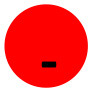
César et al. (2021)	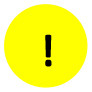	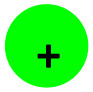	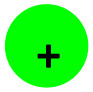	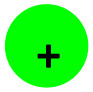	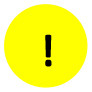	N/A	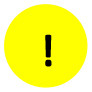
Cooke et al. (2018)	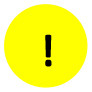	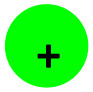	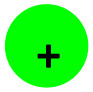	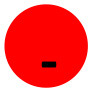	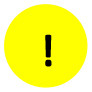	N/A	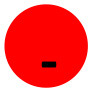
Kokkinidis et al. (1998)	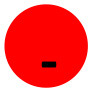	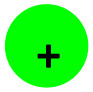	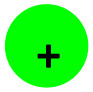	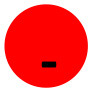	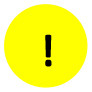	N/A	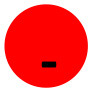
McGrath et al. (2014)	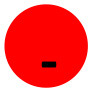	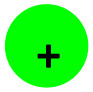	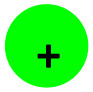	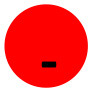	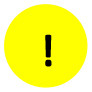	N/A	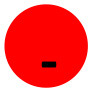
Mika et al. (2007)	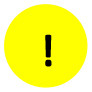	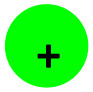	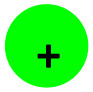	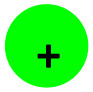	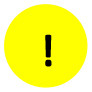	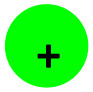	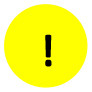
Muanjai and Namsawang (2015)	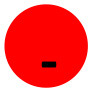	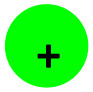	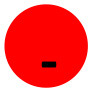	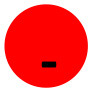	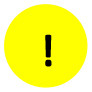	N/A	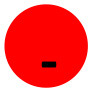
Torres et al. (2005)	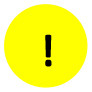	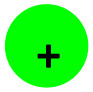	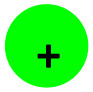	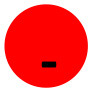	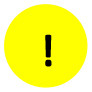	N/A	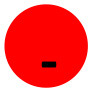
Torres et al. (2013)	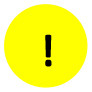	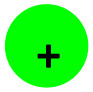	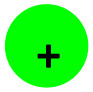	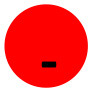	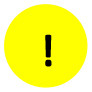	N/A	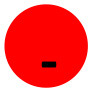
West et al. (2014)	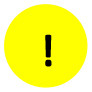	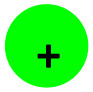	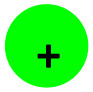	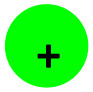	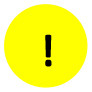	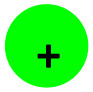	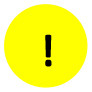

*D1, Randomization process; D2, Deviations from intended intervention, effect of assignment to intervention; D3, Missing outcome data; D4, Measurement of the outcome; D5, Selection of the reported result; DS, Domain S, Bias arising from period and crossover effects; specific to crossover designs and not applicable to parallel trials; VAS, Visual analog scale; N/A, Not applicable; Colors: green means low risk of bias; yellow means some concerns; red means high risk of bias*.

**Figure 2:**
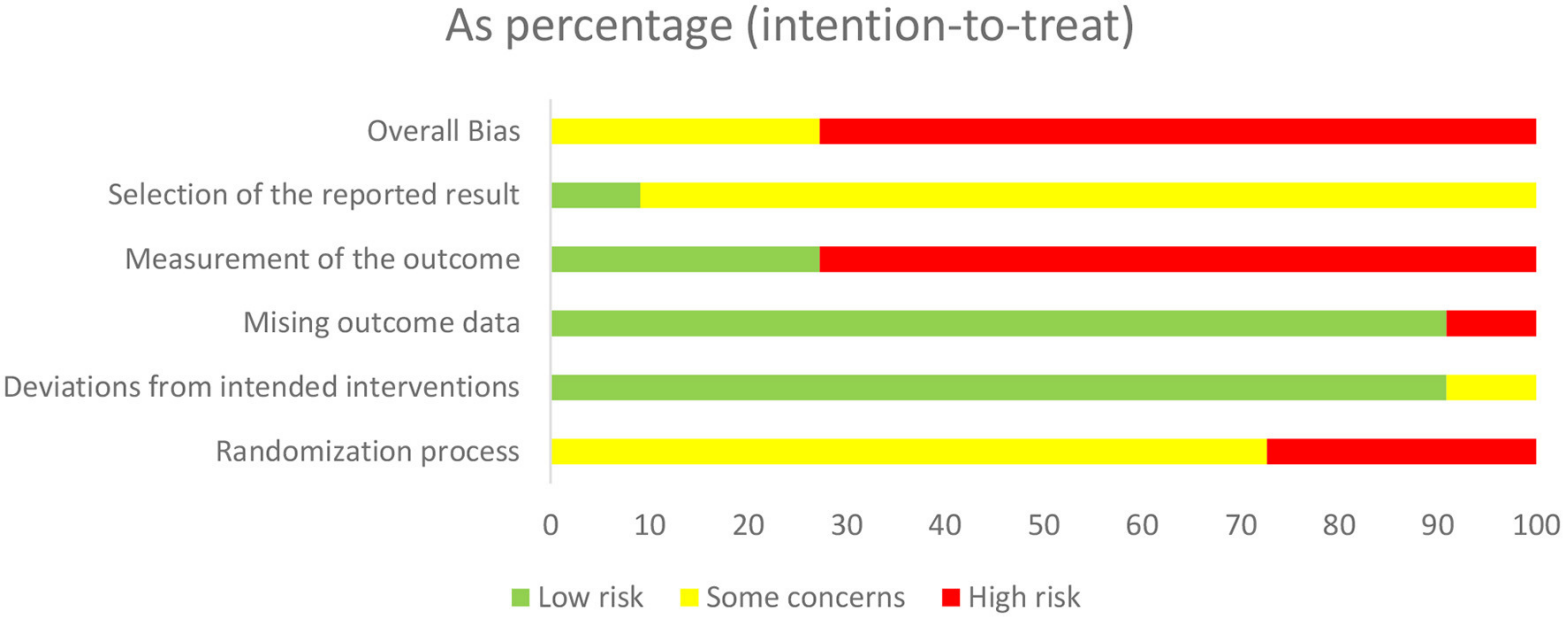
Percentage distribution of risk of bias in individual studies (RoB 2).

These results can be visualized in Figure 2, which exhibits the percentage distribution of RoB for domains 1–5 and overall bias considering the worst assessment for each study. Overall RoB was high in 72.7% of the studies and presented some concerns in 27.3%. All studies presented problems with the randomization process: no description of how randomization was achieved and whether allocation sequence was properly concealed and, in 27.3% of the studies, baseline values suggested problems with the randomization process. Moreover, 72.7% of studies had high RoB in measurement of the outcome, mostly because testers were usually not blinded, and some outcomes were particularly prone to being influenced by knowledge of the intervention received.

There was low RoB arising from deviations from intended interventions and from missing outcome data in 90.9% of the papers. Finally, although 90.9% of papers presented some concerns for RoB arising from selection of the reported result, this resulted mostly from lack of pre-registered protocols, and our opinion upon reading the studies is that the authors provided an honest and complete reporting. Of the crossover studies, one had high RoB for carry-over effects (Cè et al., 2013) and, following protocol, was excluded from meta-analysis. However, it still integrated the qualitative review.

### Results of Individual Studies

Primary outcomes were registered on the form of means ± SDs, except for Cè et al. (2013), that used means ± SEM. This study, in particular, had a graph from which we felt we could not extract reliable data. Allied to the fact that this study could not enter the meta-analytical calculations, we chose not to extract the data from the graph, and only present the qualitative results provided by the authors. For values extracted from graphs (Mika et al., 2007; Bonfim et al., 2010; Muanjai and Namsawang, 2015; Cooke et al., 2018; César et al., 2021), Cronbach's Alpha values were 0.991 (means) and 0.981 (SDs). The results of individual studies are compiled in Table 4.

**Table 4:** Results of individual studies.

**References**	**Primary outcomes and timepoints post-recovery^a^**	**Intervention (pre-post)**	**Comparators (pre-post)**	**Main findings**
Bonfim et al. (2010)	DOMS assessed through perceived pain at 24, 48, and 72 h. Two methods: VAS (0–10 scale) + pain dorimeter applied to medial gastrocnemius.	VAS: Pre = 0; 24 h = 2.87 ± 2.14; 48 h^c^ = 3.47 ± 2.61; 72 h = 2.18 ± 2.04.Pain dorimeter: Pre = 8.48 ± 2.84; 24 h^c^ = 6.55 ± 2.79; 48 h = 6.89 ± 5.02; 72 h = 7.4 ± 2.39.	*Passive recovery (rest)* VAS: Pre = 0; 24 h = 2.85 ± 1.70; 48 h = 3.49 ± 1.90; 72 h = 1.47 ± 1.38. Pain dorimeter: Pre = 9.26 ± 3.55; 24 h = 5.74 ± 1.87; 48 h = 6.89 ± 2.13; 72 h = 8.4 ± 1.98.	No between-group differences. Stretching was not effective in alleviating DOMS. No reporting of adverse effects.
Cè et al. (2013)	MVC of knee extensor muscles until 60-min post-exercise, stand-and-reach test [excluded due to insufficient information].	MVC remained depressed after the exercise bout until 60' after recovery in all trials, regardless of recovery protocol. Passive stretching cannot be considered an alternative to active recovery in accelerating lactate kinetics after fatiguing exercise.*Concrete data was not extracted for this study due to reasons explained in the manuscript. Also, potential problems with carry-over effects motivated the exclusion of meta-analysis*.		MVC remained depressed after the exercise bout until 60' after recovery in all trials, regardless of recovery protocol. No difference between conditions at any time point. No reporting of adverse effects.
César et al. (2021)	HG strength and HG muscle endurance, immediately post-recovery.	Maximal HG strength: Pre = 33.56 ± 7.19; Post = 28.25 ± 8.39.HG endurance: Pre = 54.87 ± 12.56; Post = 48.43 ± 18.7.	*CWI* Maximal HG strength: Pre = 33.22 ± 4.79; Post = 35.79 ± 4.45. HG endurance: Pre = 45.36 ± 13.18; Post = 44.75 ± 13.49. *Passive recovery* Maximal HG strength: Pre = 35.62 ± 6.67; Post = 34.42 ± 4.79. HG endurance: Pre = 47.82 ± 10.42; Post = 35.25 ± 14.41.	CWI promoted regeneration of HG strength and endurance, while stretching and passive recovery did not. No reporting of adverse effects.
Cooke et al. (2018)	MVC of knee extensor and flexor muscles, perceived DOMS (0–13 scale). 24 h post-recovery.	MVC ext. 60°/s: pre-exercise = 0.33 ± 0.09; 24 h = 0.31 ± 0.08.MVC ext. 180°/s: pre-exercise = 0.31 ± 0.05; 24 h = 0.29 ± 0.09.MVC fle. 60°/s: pre-exercise = 0.21 ± 0.05; 24 h = 0.20 ± 0.05.MVC fle. 180°/s: pre-exercise = 0.16 ± 0.06; 24 h = 0.16 ± 0.06.DOMS: pre-exercise = 1.2 ± 1.67; 24 h = 7.34 ± 1.60.	*Treadmill* MVC ext. 60°/s: pre-exercise = 0.35 ± 0.08; 24 h = 0.29 ± 0.08. MVC ext. 180°/s: pre-exercise = 0.39 ± 0.24; 24 h = 0.27 ± 0.12. MVC fle. 60°/s: pre-exercise = 0.21 ± 0.05; 24 h = 0.20 ± 0.07. MVC fle. 180°/s: pre-exercise = 0.16 ± 0.03; 24 h = 0.16 ± 0.05. DOMS: pre-exercise = 0.52 ± 1.36; 24 h = 7.75 ± 1.77. *Anti-gravity treadmill* MVC ext. 60°/s: pre-exercise = 0.32 ± 0.10; 24 h = 0.27 ± 0.12. MVC ext. 180°/s: pre-exercise = 0.24 ± 0.10; 24 h = 0.22 ± 0.09. MVC fle. 60°/s: pre-exercise = 0.20 ± 0.07; 24 h = 0.17 ± 0.06. MVC fle. 180°/s: pre-exercise = 0.14 ± 0.04; 24 h = 0.14 ± 0.04. DOMS: pre-exercise = 1.44 ± 1.43; 24 h = 6.52 ± 1.84.	MVC decreased in all groups until 24 h post-exercise, while DOMS increased. No between-group differences for any outcome at any time point. No reporting of adverse effects.
Kokkinidis et al. (1998)	Muscle pain/DOMS (VAS 1–10). *1 RM knee flexion and sit-and-reach were excluded because only the mean was presented, without variation measures. 24 h post-recovery*.	DOMS: pre-exercise = 1 ± 0; 24 h = 4.8 ± 1.1.	*Passive recovery (rest)* DOMS: pre-exercise = 1 ± 0; 24 h = 5.0 ± 1.2. *Cryotherapy* DOMS: pre-exercise = 1 ± 0; 24 h = 4.3 ± 0.4.	All groups had increased DOMS at 24 h post-exercise. Stretching and cryotherapy were not effective in diminishing DOMS in comparison with passive recovery. No reporting of adverse effects.
McGrath et al. (2014)	Sit-and-reach, muscle Soreness Scale (1–6) at 24 and 48 h. Sit-and-reach also immediately post-recovery.	Sit-and-reach: pre-exercise = 6.0 ± 9.7; immediately post-exercise = 8.5 ± 9.7. DOMS: pre-exercise = 0; 24 h = 2.3 ± 1.1; 48 h = 2.0 ± 1.1	*PNF* Sit-and-reach: pre-exercise = 5.2 ± 10.2; immediately post-exercise = 6.2 ± 10.3. DOMS: pre-exercise = 0; 24 h = 2.2 ± 0.8; 48 h = 1.6 ± 1.0. *Passive recovery (rest)* Sit-and-reach: pre-exercise = 7.9 ± 9.7; immediately post-exercise = 9.0 ± 9.4. DOMS: pre-exercise = 0; 24 h = 1.9 ± 0.9; 48 h = 1.7 ± 0.9.	Stretching group had no significant decrease in DOMS at 24 and 48 h, while the comparator groups had at 48 h. Static stretching impaired recovery in comparison with PNF and passive recovery. No reporting of adverse effects.
Mika et al. (2007)	Isometric knee extension at 50% of MVC to the point of fatigue, static knee extension at 78°, while sitting (MedX leg-extension dynamometer), immediately post-recovery.	MVC: pre-exercise = 224.21 ± 70.43; immediately post-exercise = 205.84 ± 78.85.	*Cycling* MVC: pre-exercise = 224.21 ± 70.43; immediately post-exercise = 214.26 ± 97.99. *Passive recovery (rest)* MVC: pre-exercise = 224.21 ± 70.43; immediately post-exercise = 207.37 ± 55.12.	MVC was significantly higher after cycling than after stretching or passive recovery. No reporting of adverse effects.
Muanjai and Namsawang (2015)	Soreness sensation (0–100 VAS) during knee extensor MVC and stretching, active ROM (knee flexion), knee extensors isometric MVC at 90°, vertical jump. Immediately post-recovery, as well as at 24, 48, and 72 h^b^.	MVC: pre-exercise = 0.39 ± 0.1; post-recovery = 0.27 ± 0.04; 24 h = 0.21 ± 0.04; 48 h = 0.31 ± 0.02; 72 h = 0.35 ± 0.04. Knee flexion ROM: pre-exercise = 131.7 ± 5.5; post-recovery: 128.2 ± 3.5; 24 h = 123.9 ± 3.0; 48 h = 126.6 ± 3.0; 72 h = 129.4 ± 1.9. Vertical jump high: pre-exercise = 56.5 ± 8.9; post-recovery = 51.4 ± 5.7; 24 h = 48.4 ± 5.7; 48 h = 49.9 ± 3.3; 72 h = 51.8 ± 5.1. Soreness on MVC: pre-exercise = 0 ± 0; post-recovery = 5.33 ± 7.61; 24 h = 20.05 ± 15.48; 48 h = 14.21 ± 19.55; 72 h = 7.11 ± 9.64. Soreness on passive stretching: pre-exercise = 0 ± 0; post-recovery = 2.94 ± 3.64; 24 h = 16.62 ± 16.8; 48 h = 3.98 ± 5.2; 72 h = 3.64 ± 6.23.	*CWI* MVC: pre-exercise = 0.38 ± 0.1; post-recovery = 0.30 ± 0.04; 24 h = 0.25 ± 0.04; 48 h = 0.30 ± 0.04; 72 h = 0.35 ± 0.03. Knee flexion ROM: pre-exercise = 133.8 ± 4.4; post-recovery = 129.2 ± 3.4; 24 h = 126.3 ± 6.6; 48 h = 127.2 ± 4.7; 72 h = 130.1 ± 2.9. Vertical jump height: pre-exercise = 54.6 ± 6.3; post-recovery = 43.4 ± 5.5; 24 h = 45.0 ± 5.1; 48 h = 46.9 ± 4.9; 72 h = 50.0 ± 4.2. Soreness on MVC: pre-exercise = 0 ± 0; post-recovery = 8.63 ± 11.93; 24 h = 25.89 ± 25.89; 48 h = 17.13 ± 17.39; 72 h = 10.15 ± 13.20. Soreness on passive stretching: pre-exercise = 0 ± 0; post-recovery = 12.99 ± 21.99; 24 h = 15.93 ± 20.43; 48 h = 15.76 ± 22.85; 72 h = 8.83 ± 15.59.	For both groups, soreness increased after exercise, peaked at 24 h, and gradually returned to baseline levels at 96 h. MVC of knee extensors had the lowest peak value at 24 h, having returned to baseline at 48 h. Vertical jump started recovering immediately post-exercise, but was not back to baseline even at 96 h. No differences between groups. Explicit statement reporting there were no adverse effects.
Torres et al. (2005)	DOMS through perceived pain (VAS), maximal eccentric peak torque (knee extensors). 1, 24, 48, and 72 h^b^.	Maximal eccentric peak torque: pre-exercise = 303.1 ± 59.96; 1 h = 231.1 ± 49.1; 24 h = 266.5 ± 57.5; 48 h = 275.3 ± 54.2; 72 h = 285.7 ± 63.7.DOMS: pre-exercise = 0.0 ± 0.0; 1 h = 0.6 ± 0.7; 24 h = 4.1 ± 1.2; 48 h = 5.7 ± 1.8; 72 h = 3.6 ± 2.0.	*Passive recovery (rest)* Maximal eccentric peak torque: pre-exercise = 352.6 ± 76.49; 1 h = 276.1 ± 66.3; 24 h = 294.9 ± 65.7; 48 h = 308.2 ± 65.5; 72 h = 318.1 ± 75.1. DOMS: pre-exercise = 0.0 ± 0.0; 1 h = 0.6 ± 0.5; 24 h = 3.2 ± 0.6; 48 h = 5.3 ± 1.5; 72 h = 2.8 ± 1.6.	DOMS began in the first hour post-exercise, achieved a peak at 48 h, and pain to palpation was still present at 96 h. No differences between the groups at any time point. No reporting of adverse effects.
Torres et al. (2013)	Muscle soreness (VAS), maximal concentric peak torque (knee extensors). 1, 24, 48, and 72 h^b^.	Muscle soreness: pre-exercise = 0.0 ± 0.0; 1 h = 1.2 ± 0.7; 24 h = 2.3 ± 1.1; 48 h = 3.5 ± 1.4; 72 h = 1.8 ± 1.3. Maximal concentric peak torque (60°/s): pre-exercise = 216.9 ± 33.5; 1 h = 173.4 ± 33.7; 24 h = 183.2 ± 42.5; 48 h = 189.7 ± 44.5; 72 h = 204.7 ± 42.6.	*Passive recovery (rest)* Muscle soreness: pre-exercise = 0.0 ± 0.0; 1 h = 0.1 ± 0.4; 24 h = 2.3 ± 0.8; 48 h = 3.8 ± 1.8; 72 h = 1.8 ± 1.2. Maximal concentric peak torque (60°/s): pre-exercise = 221.3 ± 16.7; 1 h = 181.7 ± 30.7; 24 h = 186.8 ± 27.0; 48 h = 188.5 ± 40.3; 72 h = 203.8 ± 29.3.	Significant reduction in maximal concentric peak torque and significant increases in muscle soreness. No differences between groups at any time points. No reporting of adverse effects.
West et al. (2014)	Peak power output, mean power output, time to peak power and rate to fatigue (supramaximal 30-s cycle ergometer test) at 24 h post-exercise.	Peak power: pre-exercise = 1,323 ± 323; 24 h = 1,431 ± 429. Mean power: pre-exercise = 731 ± 114.2; 24 h = 718.5 ± 125.7. Time to peak: pre-exercise = 4.68 ± 0.83; 24 h = 4.43 ± 0.49. Rate to fatigue: pre-exercise = 36.17 ± 12.5; 24 h = 40.71 ± 15.5.	*Anti-gravity treadmill* Peak power: pre-exercise = 1,323 ± 323; 24 h = 1,372 ± 364. Mean power: pre-exercise = 731 ± 114.2; 24 h = 707.7 ± 115.8. Time to peak: pre-exercise = 4.68 ± 0.83; 24 h = 4.59 ± 0.70. Rate to fatigue: pre-exercise = 36.17 ± 12.5; 24 h = 38.72 ± 12.8 *Cycle ergometer* Peak power: pre-exercise = 1,323 ± 323; 24 h = 1,411 ± 355. Mean power: pre-exercise = 731 ± 114.2; 24 h = 705.3 ± 127.6. Time to peak: pre-exercise = 4.68 ± 0.83; 24 h = 4.38 ± 0.53. Rate to fatigue: pre-exercise = 36.17 ± 12.5; 24 h = 40.15 ± 12.3.	In all groups, no differences in relation to baseline. No differences between groups. No reporting of adverse effects.

*CWI, Cold-water immersion; DOMS, Delayed onset muscular soreness (more is worse); HG, Handgrip; MVC, Maximum voluntary contraction; ROM, Range of motion; VAS, Visual Analog Scale (greater values mean worse outcomes)*.

a *As defined in our protocol*.

b *96 h not considered, as per protocol*.

Primary outcomes were any assessments related to strength, ROM and/or soreness, both short-term (i.e., until ≤ 1-h post-recovery) and delayed (24, 48, and 72 h post-recovery). These outcomes were useful only if there were pre-exercise and post-recovery assessments. Short-term effects were reported for strength-related measures in six studies (Torres et al., 2005, 2013; Mika et al., 2007; Cè et al., 2013; Muanjai and Namsawang, 2015; César et al., 2021), ROM in one study (McGrath et al., 2014; Muanjai and Namsawang, 2015), and DOMS in three studies (Torres et al., 2005, 2013; Muanjai and Namsawang, 2015). Three studies had no short-term assessments (Kokkinidis et al., 1998; Bonfim et al., 2010; West et al., 2014). One study mentioned having data at 15- and 30-min after recovery, but that data only applied to secondary outcomes (Cooke et al., 2018). With the exception of César et al. (2021), all strength-related assessments were performed for the lower limbs, and this was valid also for delayed assessments.

Delayed assessments were performed for strength-related variables in five studies (Torres et al., 2005, 2013; West et al., 2014; Muanjai and Namsawang, 2015; Cooke et al., 2018), ROM in one (Muanjai and Namsawang, 2015), and DOMS in seven (Kokkinidis et al., 1998; Torres et al., 2005, 2013; Bonfim et al., 2010; McGrath et al., 2014; Muanjai and Namsawang, 2015; Cooke et al., 2018). Three studies did not have delayed outcomes (Mika et al., 2007; Cè et al., 2013; César et al., 2021). Although Kokkinidis et al. (1998) assessed delayed effects on strength and ROM, they presented only means, without any measure of variation that could help to better interpret the results. As previously explained, if the delayed assessments were conducted after a new bout of the recovery protocol, they would be discarded, as the effects of the first bout could no longer be assessed. Of the studies including delayed assessments, four had data for the three timepoints defined in our protocol (i.e., 24, 48, and 72 h) (Torres et al., 2005, 2013; Bonfim et al., 2010; Muanjai and Namsawang, 2015), one study had data for 24 and 48 h post-recovery protocol (McGrath et al., 2014), and three had data for 24 h post-recovery only (Kokkinidis et al., 1998; West et al., 2014; Cooke et al., 2018).

Based on their data, some studies concluded that post-exercise stretching was not an effective recovery strategy, and was not superior to comparator interventions (West et al., 2014; Cooke et al., 2018), including passive recovery, i.e., rest (Bonfim et al., 2010; Cè et al., 2013; César et al., 2021). In the study of Kokkinidis et al. (1998), the authors stated that stretching and cryotherapy were superior to passive rest, but these effects were not observed at 24 h, only at 48 h; moreover, after 24 h, the experimental groups had an additional recovery bout applied, but without the soreness-inducing exercise. In study of McGrath et al. (2014), PNF was not superior to passive recovery, and the static stretching group was the only one not showing significant decreases in DOMS at 24 or 48 h.

In the study of Mika et al. (2007), short-term strength levels recovered faster in the low-intensity cycling group than in the stretching or passive rest groups. In two studies (Torres et al., 2005, 2013), the authors stated that post-exercise stretching did not impair recovery in terms of strength and DOMS when compared to a passive rest group, but it did not improve recovery either. Finally, Muanjai and Namsawang (2015) concluded that both stretching and cold-water immersion could be used to improve post-exercise recovery. However, this conclusion is not sustained on their data, as DOMS only returned to baseline at 96 h post-recovery protocol, strength levels and ROM after 48 h, and vertical jump was still not back to baseline even after 96 h. Moreover, without a passive recovery group to compare to, no statement can be provided regarding acceleration of recovery.

### Synthesis of Results

As stipulated in the protocol, cross-over trials would only be combined with parallel trials if there were no significant carryover effects (Elbourne et al., 2002). This was not guaranteed in the study of Cè et al. (2013), which was therefore excluded from meta-analysis. Across the remaining nine studies, as previously presented, there was considerable variation concerning the soreness-inducing protocols, the comparators to stretching, the outcome domains, the measurements within those outcome domains, and the timepoints of assessing the outcomes. Our protocol had stipulated three primary outcomes (strength, ROM, and DOMS) across four different timepoints (short-term, i.e., maximum 1 h after the recovery intervention; and 24, 48, and 72 h after the recovery intervention). After analyzing the outcomes and timepoints in each study, and also considering the comparator protocols, we found that only a few meta-analytical comparisons were feasible.

#### Short-Term Effects on Strength, Stretching vs. Passive Recovery (Rest)

Three studies had comparable data (i.e., strength measures of the knee extensors) to afford this meta-analysis (Torres et al., 2005, 2013; Mika et al., 2007). One study used PNF stretching (Mika et al., 2007) and the others used passive static stretching and compared this intervention to passive rest. Although the study of César et al. (2021) had strength assessments, they were for the upper limbs, more specifically grip strength, and so we decided not to compare it with the remaining studies. In RoB assessments considering this outcome, these studies had an overall classification of “some concerns,” meaning none of the domains presented high RoB. In domain 4 (measurement of the outcome), they had low RoB.

**Figure 3:**
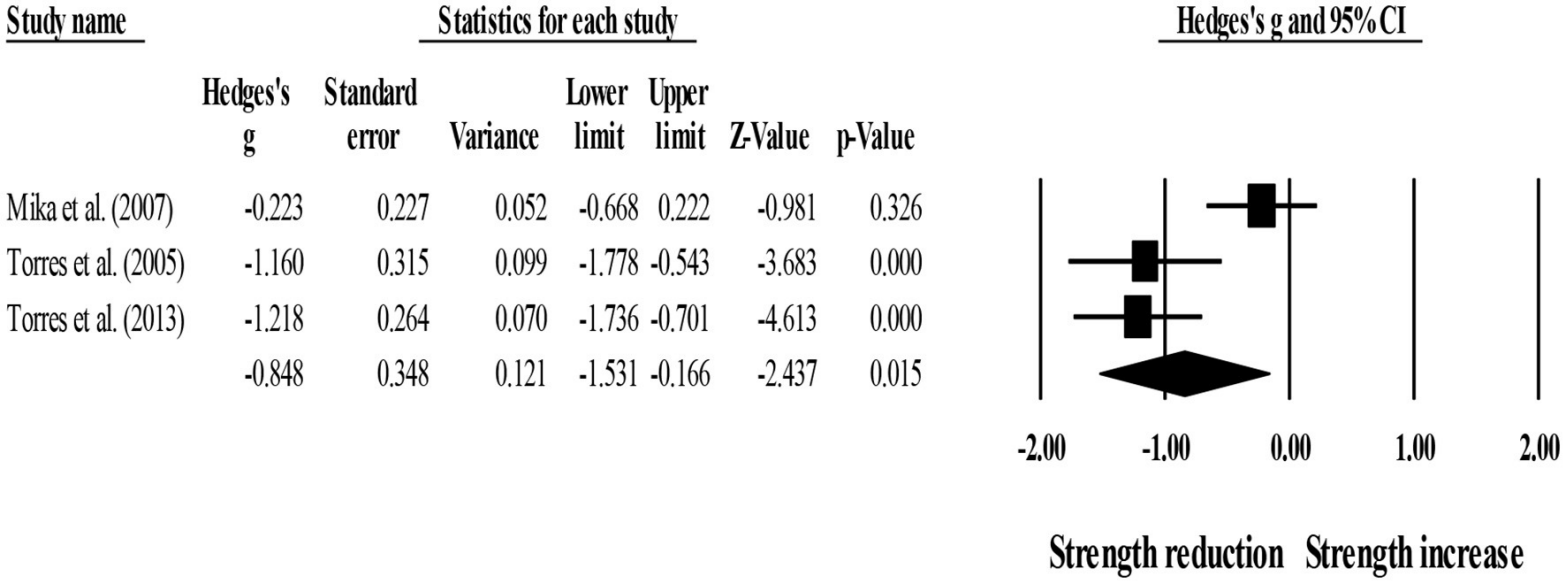
Forest plot denoting short-term strength recovery level in participants that completed post-exercise stretching protocols. Values shown are effect sizes (Hedges's g) with 95% confidence intervals (CI). The size of the plotted squares reflects the statistical weight of each study. The black diamond reflects the overall result. Note: negative values denote that post-exercise stretching protocols did not allow participants to recover their basal strength level (i.e., 0.00 in the figure).

For within-group effects, three studies provided data for short-term strength recovery, involving three stretching groups (pooled *n* = 33). Results showed that post-exercise stretching protocols did not allow participants to recover their basal strength level (ES = −0.85; 95% CI = −1.53 to −0.17; *p* = 0.015; *I*^2^ = 80.4%; Egger's test *p* = 0.396; Figure 3).

**Figure 4:**
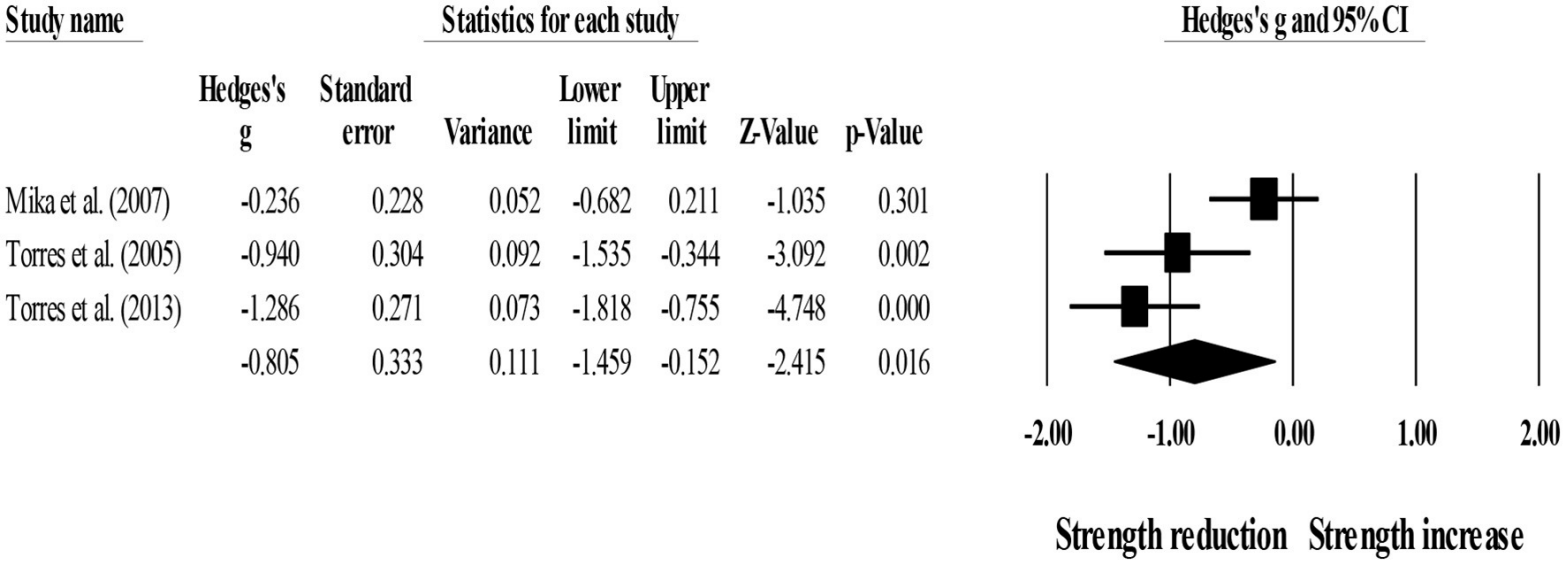
Forest plot denoting short-term strength recovery level in participants that completed post-exercise passive recovery protocols. Values shown are effect sizes (Hedges's g) with 95% confidence intervals (CI). The size of the plotted squares reflects the statistical weight of each study. The black diamond reflects the overall result. Note: negative values denote that post-exercise passive recovery protocols did not allow participants to recover their basal strength level (i.e., 0.00 in the figure).

In addition, three studies provided data for short-term strength recovery, involving three passive recovery groups (pooled *n* = 32). Results showed that post-exercise passive recovery protocols did not allow participants to recover their basal strength level (ES = −0.81; 95% CI = −1.46 to −0.15; *p* = 0.016; *I*^2^ = 78.7%; Egger's test *p* = 0.435; Figure 4).

**Figure 5:**
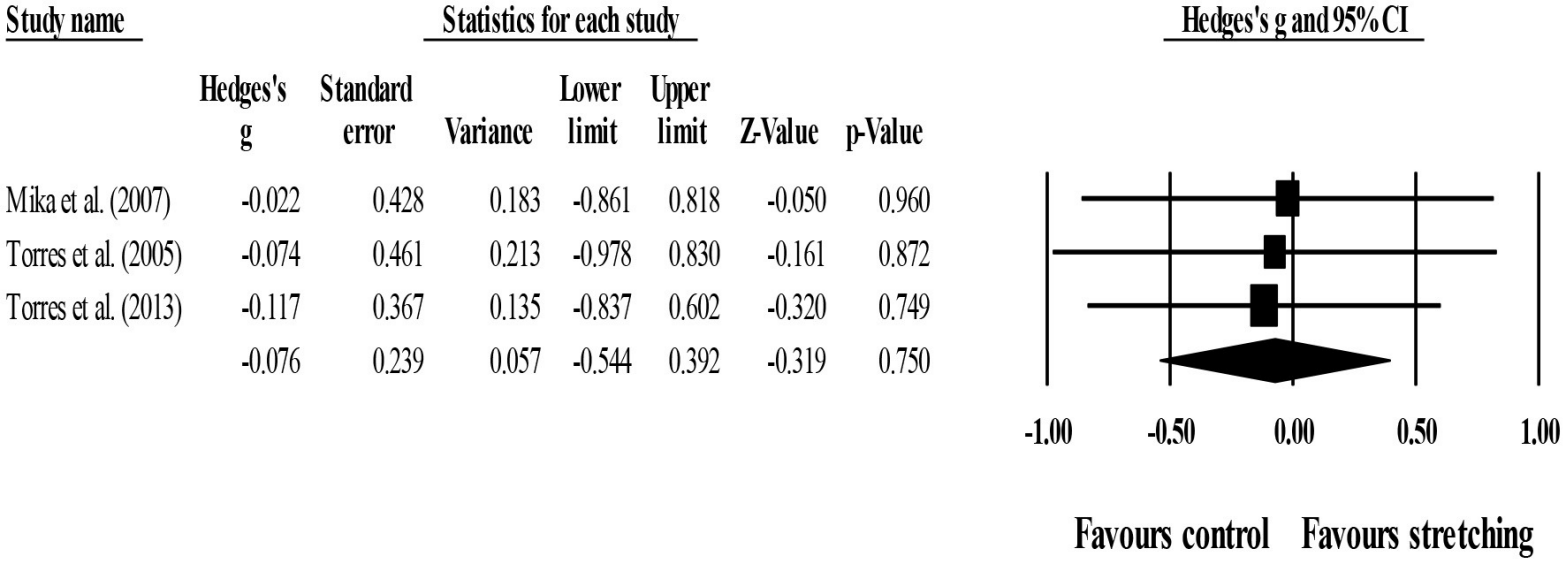
Forest plot of changes in short-term strength recovery after participating in post-exercise stretching protocols compared to control conditions (i.e., passive recovery). Values shown are effect sizes (Hedges's g) with 95% confidence intervals (CI). The size of the plotted squares reflects the statistical weight of each study. The black diamond reflects the overall result.

Between-group comparisons (pooled *n* = 65) showed no effect of post-exercise stretching protocols on strength recovery (ES = −0.08; 95% CI = −0.54 to 0.39; *p* = 0.750; *I*^2^ = 0.0%; Egger's test *p* = 0.531; Figure 5) when compared to control condition (i.e., passive recovery).

#### Delayed Effects (24 h) on Delayed Onset Muscle Soreness, Stretching vs. Passive Recovery (Rest)

Five studies had comparable data to assess DOMS at 24 h (Kokkinidis et al., 1998; Torres et al., 2005, 2013; Bonfim et al., 2010; McGrath et al., 2014). Two used active static stretching (Kokkinidis et al., 1998; Bonfim et al., 2010) and three passive static stretching (Torres et al., 2005, 2013; McGrath et al., 2014). All had at least one comparator that passively recovered (i.e., rest). The study of Bonfim et al. (2010) had two assessments of DOMS; here, we used the assessment through the visual analog scale, as the other studies also used similar scales. The four studies had high RoB in measurement of this outcome, so all results should be considered with caution.

**Figure 6:**
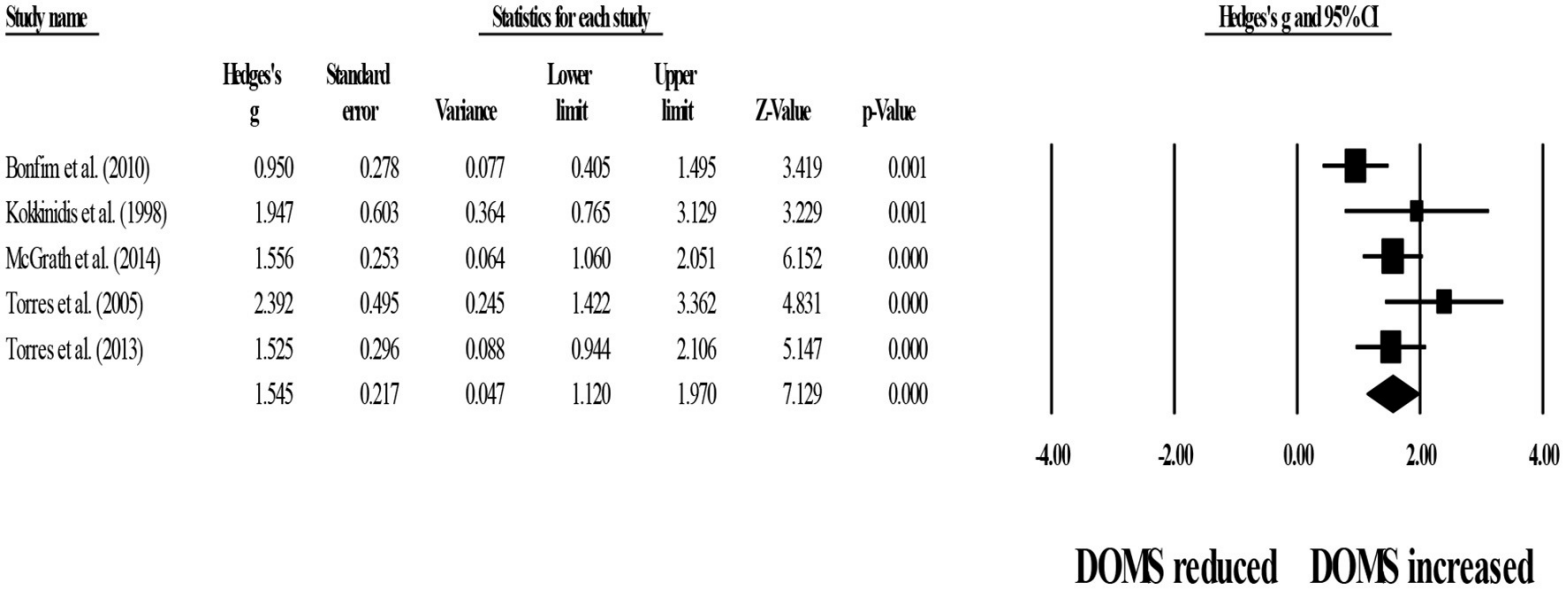
Forest plot denoting 24-h post-exercise delayed onset of muscle soreness (DOMS) in participants that completed post-exercise stretching protocols. Values shown are effect sizes (Hedges's g) with 95% confidence intervals (CI). The size of the plotted squares reflects the statistical weight of each study. The black diamond reflects the overall result. Note: positive values denote that post-exercise stretching protocols did not allow participants to recover their basal DOMS level (i.e., 0.00 in the figure).

For within-group comparisons, five studies provided data for 24-h post-exercise DOMS, involving five experimental groups (pooled *n* = 57). Results showed that post-exercise DOMS remained significantly above basal levels after post-exercise stretching protocols (ES = 1.55; 95% CI = 1.12–1.97; *p* < 0.001; *I*^2^ = 48.3%; Egger's test *p* = 0.231; Figure 6).

**Figure 7:**
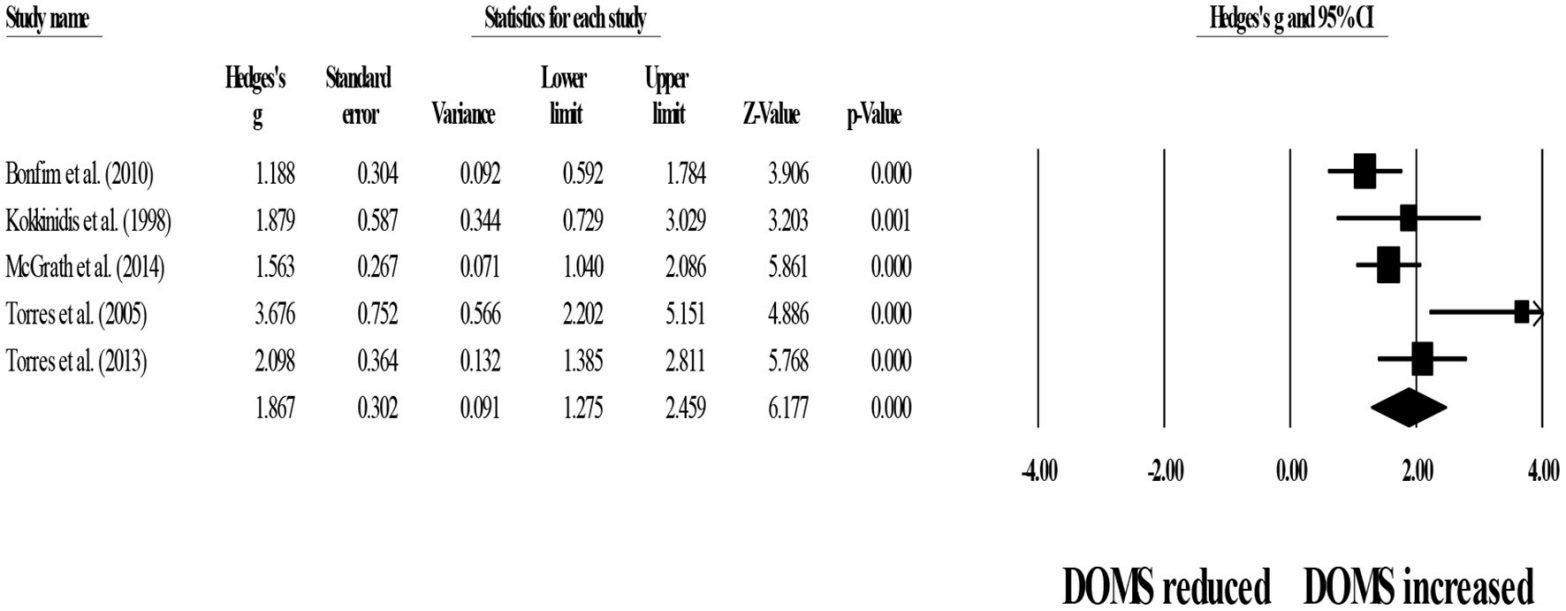
Forest plot denoting 24-h post-exercise delayed onset of muscle soreness (DOMS) in participants that completed passive recovery (control conditions) protocols. Values shown are effect sizes (Hedges's g) with 95% confidence intervals (CI). The size of the plotted squares reflects the statistical weight of each study. The black diamond reflects the overall result. Note: positive values denote that passive recovery protocols did not allow participants to recover their basal DOMS level (i.e., 0.00 in the figure).

In addition, five studies provided data for 24-h post-exercise DOMS, involving five control groups (pooled *n* = 54). Results showed that passive recovery protocols did not allow participants to recover their basal DOMS level (ES = 1.87; 95% CI = 1.28–2.46; *p* < 0.001; *I*^2^ = 64.6%; Egger's test *p* = 0.119; Figure 7).

**Figure 8:**
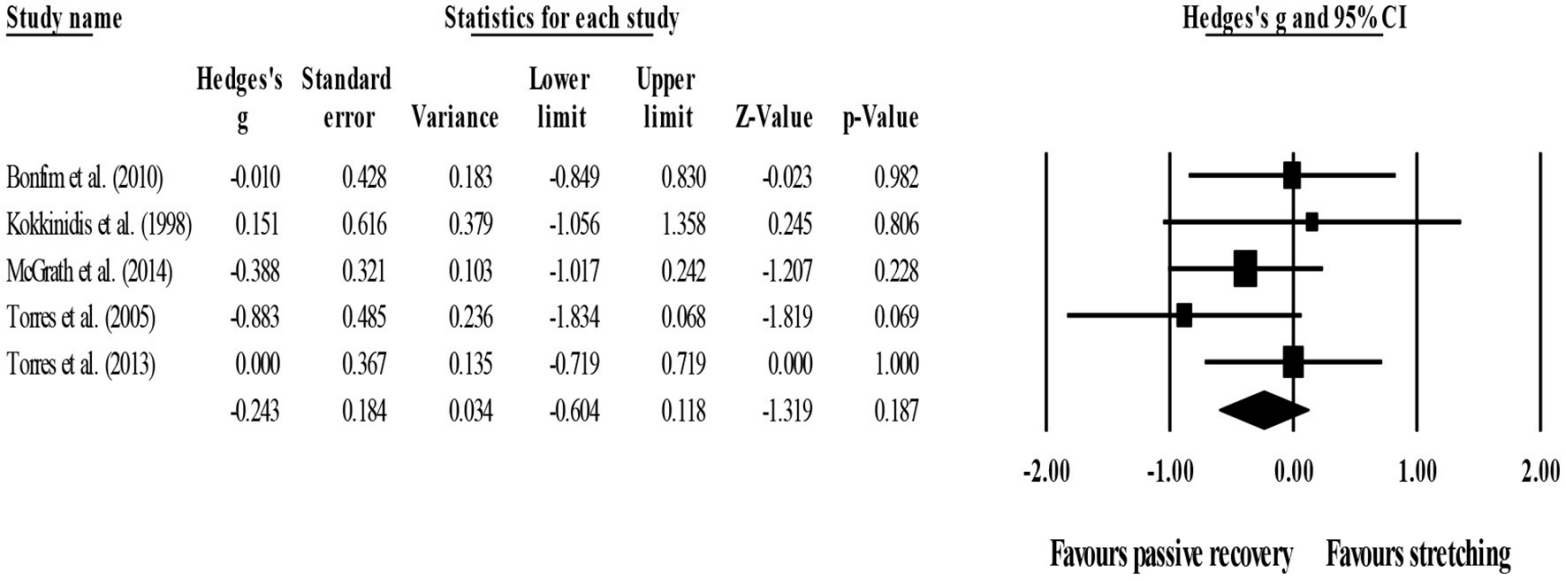
Forest plot of changes in 24-h post-exercise delayed onset of muscle soreness (DOMS) after participating in post-exercise stretching protocols compared to control conditions (i.e., passive recovery). Values shown are effect sizes (Hedges's g) with 95% confidence intervals (CI). The size of the plotted squares reflects the statistical weight of each study. The black diamond reflects the overall result.

Between-group comparisons involved five experimental and five control groups (pooled *n* = 111). Results showed no effect of post-exercise stretching protocols on 24-h post-exercise DOMS (ES = −0.24; 95% CI = −0.60–0.12; *p* = 0.187; *I*^2^ = 0.0%; Egger's test *p* = 0.880; Figure 8) when compared to control conditions (i.e., passive recovery).

#### Delayed Effects (48 h) on Delayed Onset Muscle Soreness, Stretching vs. Passive Recovery (Rest)

Four studies had comparable data (Torres et al., 2005, 2013; Bonfim et al., 2010; McGrath et al., 2014). One used active static stretching (Bonfim et al., 2010) and three passive static stretching (Torres et al., 2005, 2013; McGrath et al., 2014). All had at least one comparator that passively recovered (i.e., rest). With regard to RoB, four studies had high RoB in measurement of this outcome.

**Figure 9:**
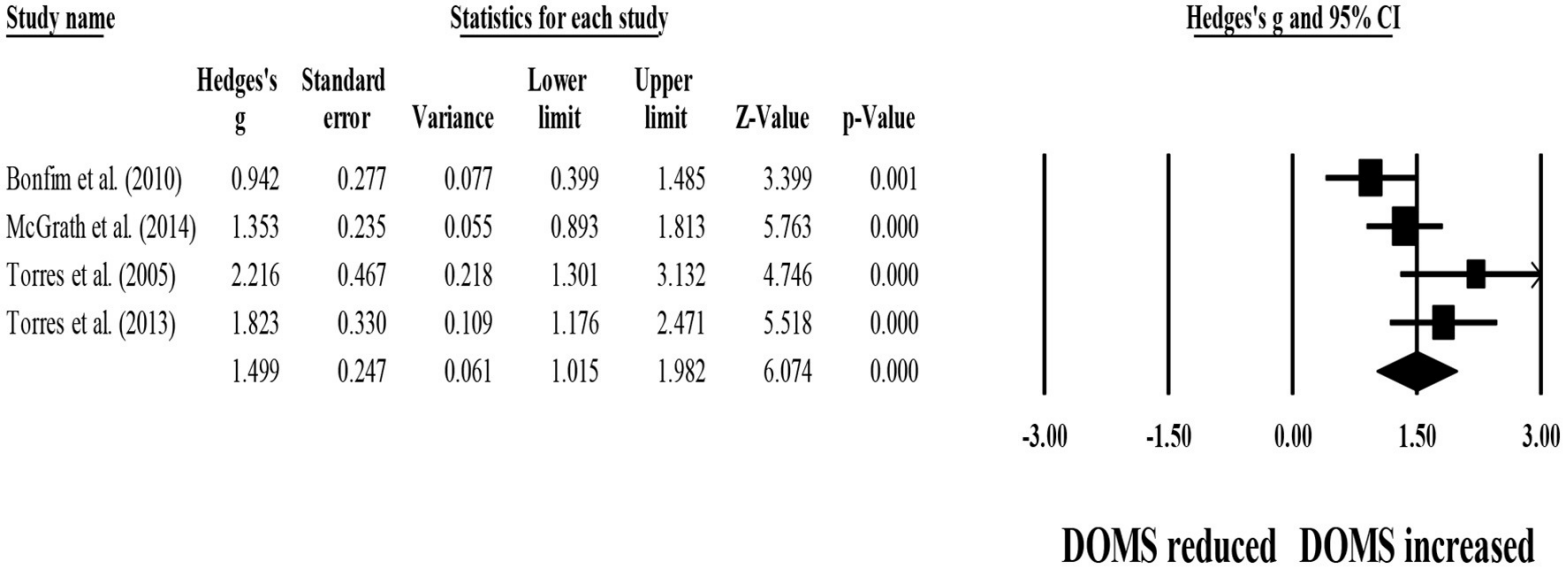
Forest plot denoting 48-h post-exercise delayed onset of muscle soreness (DOMS) in participants that completed post-exercise stretching protocols. Values shown are effect sizes (Hedges's g) with 95% confidence intervals (CI). The size of the plotted squares reflects the statistical weight of each study. The black diamond reflects the overall result. Note: positive values denote that post-exercise stretching protocols did not allow participants to recover their basal DOMS level (i.e., 0.00 in the figure).

Four studies provided data for within-group comparisons on 48-h post-exercise DOMS, involving four experimental groups (pooled *n* = 53). Results showed that post-exercise DOMS remained significantly above basal levels after post-exercise stretching protocols (ES = 1.50; 95% CI = 1.02–1.98; *p* < 0.001; *I*^2^ = 59.8%; Egger's test *p* = 0.257; Figure 9).

**Figure 10:**
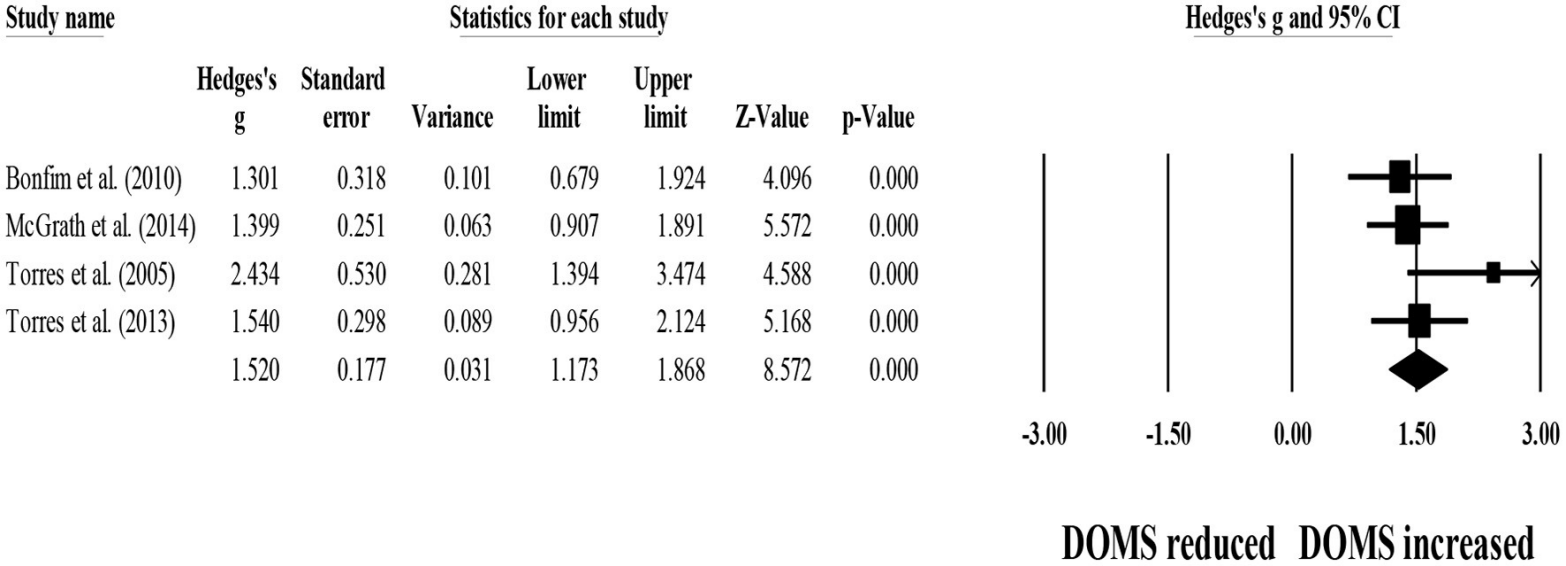
Forest plot denoting 48-h post-exercise delayed onset of muscle soreness (DOMS) in participants that completed post-exercise passive recovery. Values shown are effect sizes (Hedges's g) with 95% confidence intervals (CI). The size of the plotted squares reflects the statistical weight of each study. The black diamond reflects the overall result. Note: positive values denote that post-exercise passive recovery did not allow participants to recover their basal DOMS level (i.e., 0.00 in the figure).

Four studies provided data for 48-h post-exercise DOMS, involving four control groups (i.e., passive recovery) (pooled *n* = 50). Results showed that post-exercise passive recovery protocols did not allow participants to recover their basal DOMS level (ES = 1.52; 95% CI = 1.17–1.87; *p* < 0.001; *I*^2^ = 18.3%; Egger's test *p* = 0.120; Figure 10).

**Figure 11:**
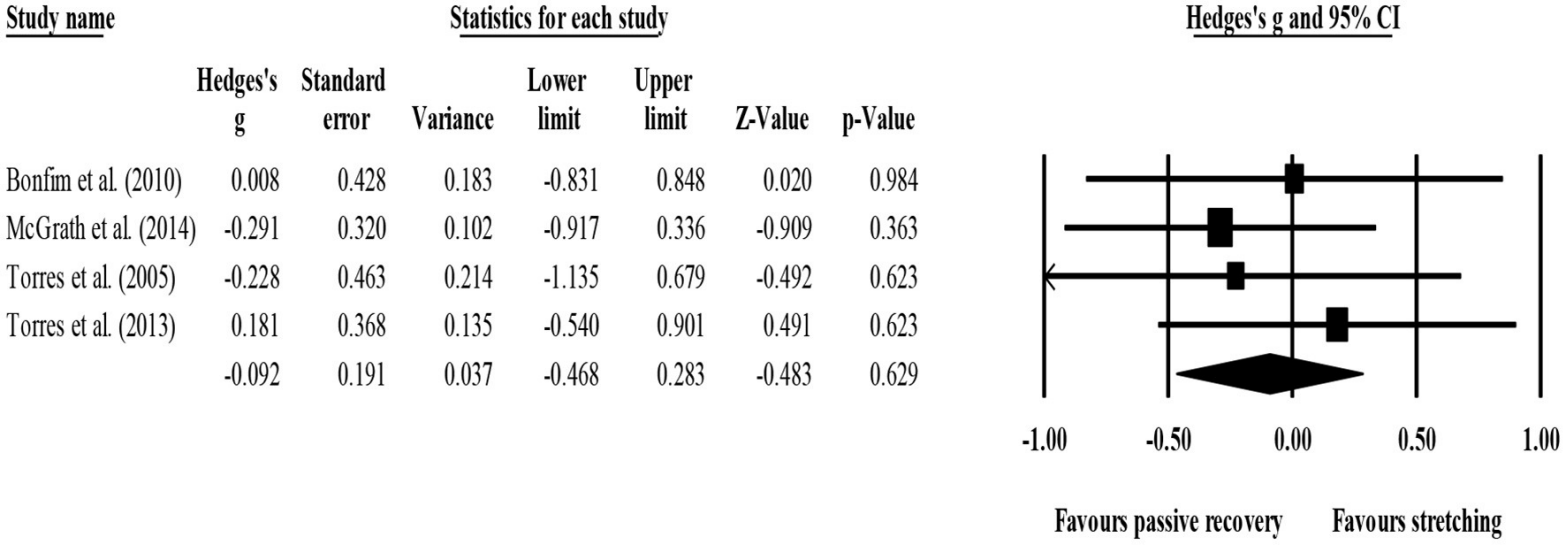
Forest plot of changes in 48-h post-exercise delayed onset of muscle soreness (DOMS) after participating in post-exercise stretching protocols compared to control conditions (i.e., passive recovery). Values shown are effect sizes (Hedges's g) with 95% confidence intervals (CI). The size of the plotted squares reflects the statistical weight of each study. The black diamond reflects the overall result.

For between-group comparisons, four studies provided data for 48-h post-exercise DOMS, involving four experimental and four control groups (pooled *n* = 103). Results showed no effect of post-exercise stretching protocols on 48-h post-exercise DOMS (ES = −0.09; 95% CI = −0.47–0.28; *p* = 0.629; *I*^2^ = 0.0%; Egger's test *p* = 0.777; Figure 11) when compared to control conditions (i.e., passive recovery).

#### Delayed Effects (72 h) on Delayed Onset Muscle Soreness, Stretching vs. Passive Recovery (Rest)

Three studies had comparable data for DOMS at 72 h (Torres et al., 2005, 2013; Bonfim et al., 2010). One used active static stretching (Bonfim et al., 2010) and two passive static stretching (Torres et al., 2005, 2013). With regard to RoB, the three studies had high RoB in measurement of this outcome.

**Figure 12:**
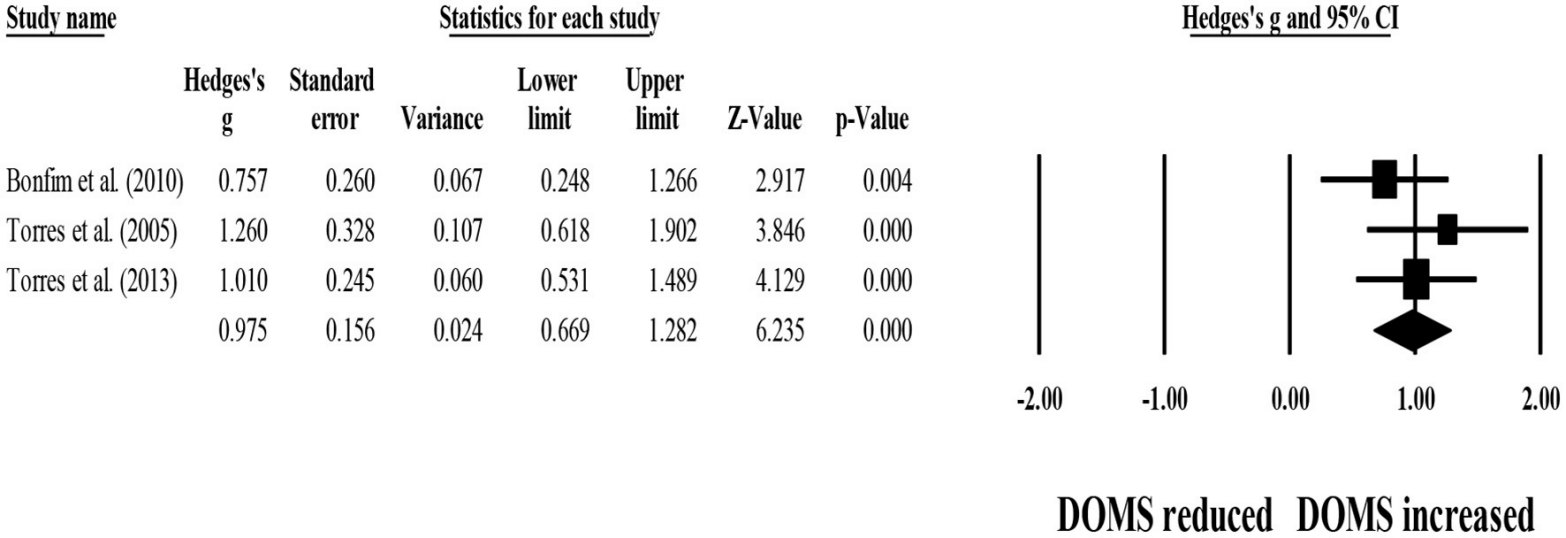
Forest plot denoting 72-h post-exercise delayed onset of muscle soreness (DOMS) in participants that completed post-exercise stretching protocols. Values shown are effect sizes (Hedges's g) with 95% confidence intervals (CI). The size of the plotted squares reflects the statistical weight of each study. The black diamond reflects the overall result. Note: positive values denote that post-exercise stretching protocols did not allow participants to recover their basal DOMS level (i.e., 0.00 in the figure).

For within-group analysis, three studies provided data for 72-h post-exercise DOMS, involving three experimental groups (pooled *n* = 33). Results showed that post-exercise DOMS remained significantly above basal levels after post-exercise stretching protocols (ES = 0.98; 95% CI = 0.67–1.28; *p* < 0.001; *I*^2^ = 0.0%; Egger's test *p* = 0.525; Figure 12).

**Figure 13:**
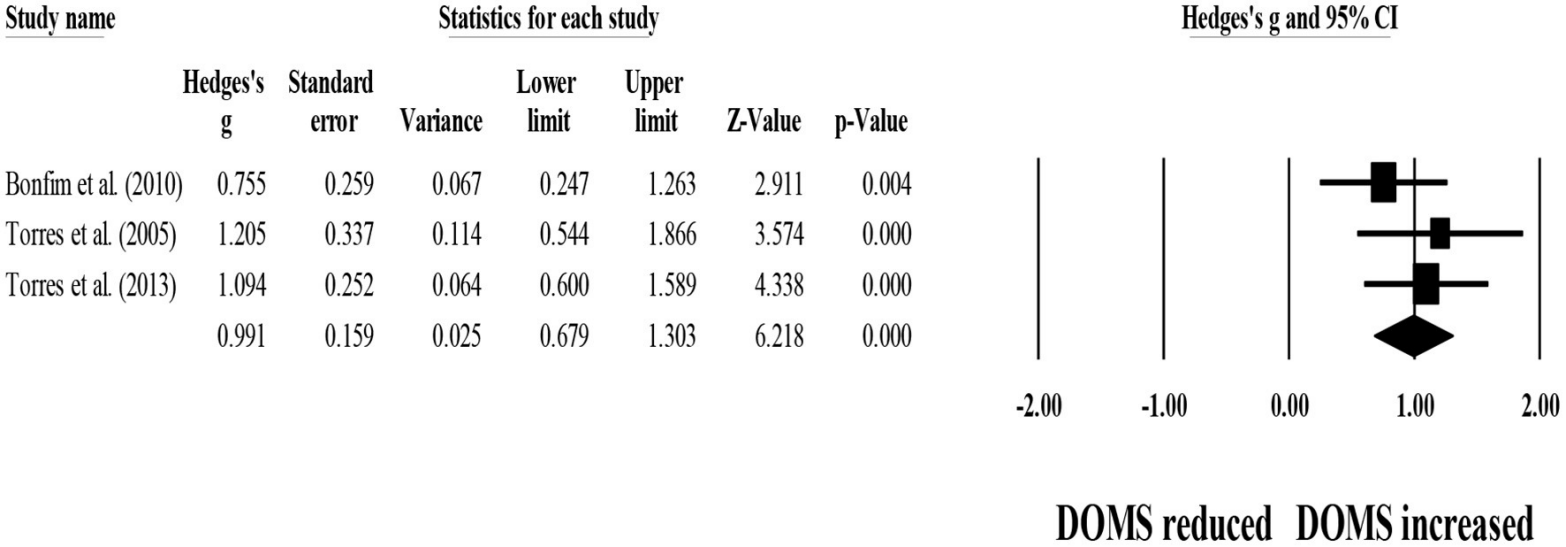
Forest plot denoting 72-h post-exercise delayed onset of muscle soreness (DOMS) in participants that completed post-exercise passive recovery protocols. Values shown are effect sizes (Hedges's g) with 95% confidence intervals (CI). The size of the plotted squares reflects the statistical weight of each study. The black diamond reflects the overall result. Note: positive values denote that post-exercise passive recovery protocols did not allow participants to recover their basal DOMS level (i.e., 0.00 in the figure).

Three studies provided data for 72-h post-exercise DOMS, involving three passive recovery groups (pooled *n* = 32). Results showed that post-exercise passive recovery protocols did not allow participants to recover their basal DOMS level (ES = 0.99; 95% CI = 0.68–1.30; *p* < 0.001; *I*^2^ = 0.0%; Egger's test *p* = 0.641; Figure 13).

**Figure 14:**
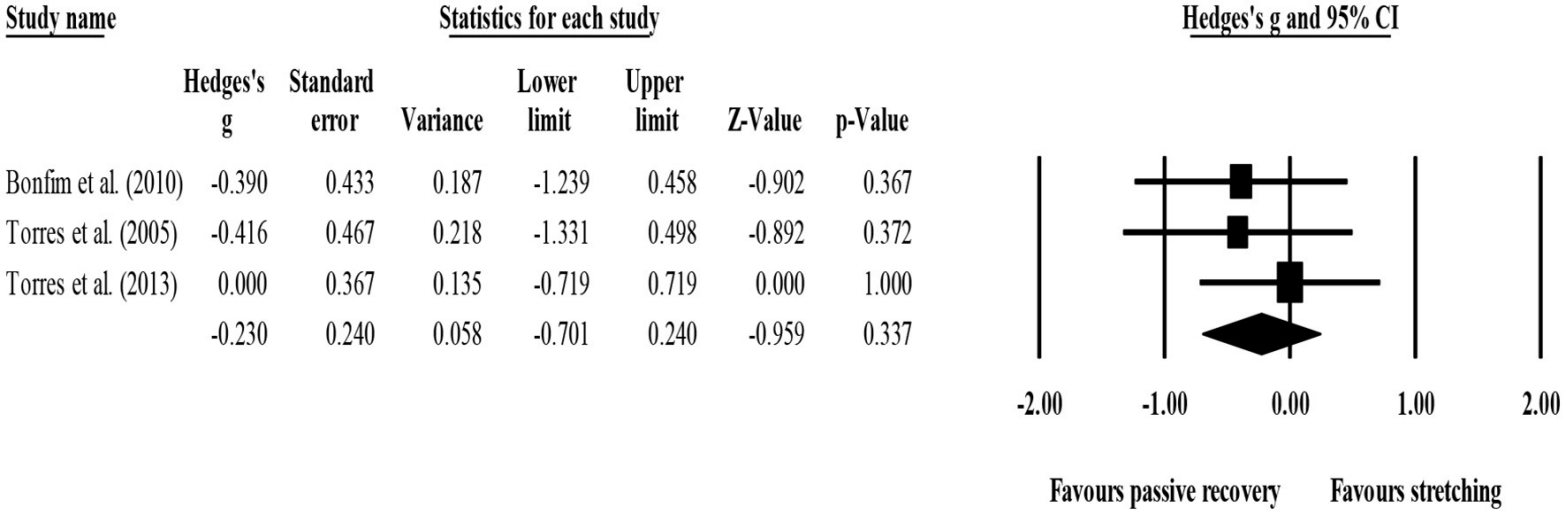
Forest plot of changes in 72-h post-exercise delayed onset of muscle soreness (DOMS) after participating in post-exercise stretching protocols compared to control conditions (i.e., passive recovery). Values shown are effect sizes (Hedges's g) with 95% confidence intervals (CI). The size of the plotted squares reflects the statistical weight of each study. The black diamond reflects the overall result.

For between-group comparisons, three studies provided data for 72-h post-exercise DOMS, involving three experimental and three control groups (pooled *n* = 65). Results showed no effect of post-exercise stretching protocols on 72-h post-exercise DOMS (ES = −0.23; 95% CI = −0.70–0.24; *p* = 0.337; *I*^2^ = 0.0%; Egger's test *p* = 0.165; Figure 14) when compared to control conditions (i.e., passive recovery).

### Additional Analysis

Due to the small number of studies included in each meta-analysis, additional analysis, and sensitivity analyses were not performed. In each analysis, RoB was similar in all studies, and so we decided not to assess the effects of RoB on the results. Meta-regression was not performed due to having <10 studies with sufficient commonalities.

### Confidence in Cumulative Evidence

Confidence in cumulative is equivalent to quality of the evidence (Higgins et al., 2019). GRADE assessments are presented in Table 5. Overall, we have very little confidence in the effect estimate, and the true effect is likely to be substantially different from the estimate of effect.

**Table 5:** GRADE assessment for the certainty of evidence.

**Outcomes^a^**	**Study design**	**Risk of bias in individual studies**	**Publication bias**	**Inconsistency**	**Indirectness**	**Imprecision**	**Confidence in evidence**	**Recommendation**
Strength, ROM and DOMS	11 RCTs and 289 participants.	High^b^	No publication bias detected^c^	High^d^	High^e^	High^f^	⊕ Very low.	No recommendation can be provided on the basis of existing data.

a *Outcomes were grouped as their assessments were not different*.

b *Detailed assessments in Table 3*.

c *Assessed through extended Egger's test*.

d *Assessed through I^2^, but also considering qualitative analysis from studies not included in meta-analysis. Because the outcomes are continuous variables, high heterogeneity was expected. Heterogeneity also likely emerged from very distinct study designs in terms of soreness-inducing protocols, as well as modality and dosage of post-exercise recovery protocols. Adverse effects were mostly unreported*.

e *Studies were mainly limited to sedentary or recreationally active subjects, while athletes and populations with pathologies are not included. Second, all measures provide only indirect assessments of the more complex phenomena of recovery*.

f *While some imprecision is expected due to referring to continuous variables, two additional factors weighted on this decision: small sample size and wide confidence intervals, generating uncertainty about magnitude of effect*.

*ROM, Range of motion; DOMS, Delayed onset muscular soreness; RCTs, randomized controlled trials*.

## Discussion

### Summary of Evidence

Stretching has been traditionally prescribed for the cool-down phase of training sessions, under the premise that it enhances recovery (ACSM, 2018; American Heart Association, 2020). But this premise has been questioned by previous assessments of the literature (Herbert and Gabriel, 2002; Henschke and Lin, 2011; Herbert et al., 2011). Therefore, we have conducted a systematic review with meta-analysis of supervised RCTs on the effects of post-exercise stretching on short-term (i.e., ≤ 1 h) and delayed (24, 48, and 72 h) recovery of strength levels, ROM, and DOMS. Searches were conducted in eight electronic databases post-protocol approval, on December 23 and 24 of 2020, and updated on February 16, 2021. Of the 17,050 records emerging from the searches and 25 additional records emerging from manual searches within reference lists, 11 RCTs were eligible for qualitative analysis (*n* = 289), and 10 for quantitative analyses (*n* = 280, with *n* = 229 after excluding groups not fulfilling PICOS criteria). Due to the overall small sample size, generalization to a broader population is not advised.

Active static stretching, passive stretching and PNF were used for post-exercise recovery, but no protocol adopted dynamic stretching. Overall, analysis of individual studies showed that there was no evidence that stretching enhanced recovery in comparison to passive recovery (i.e., rest) or to alternative recovery modalities, such as cycling and cold-water immersion. There was no evidence to the contrary, i.e., that stretching impaired recovery. Even for secondary outcomes, such as blood lactate and serum creatine kinase, for example, no strong case can be made for stretching accelerating or improving recovery. Furthermore, overall RoB was high, meaning that this field of research is lacking in terms of methodological design. Especially problematic was the wide use of unblinded testers, even for outcomes with greater degree of subjectivity.

Due to the diversity of outcomes and timepoints of assessments, only four meta-analytical comparisons were possible, all between stretching and passive recovery (i.e., rest): strength levels at ≤ 1 h, and DOMS at 24, 48, and 72 h. Overall, stretching was no more effective than passive recovery in returning strength levels and DOMS to baseline values. Heterogeneity of the meta-analysis (*I*^2^) was high for within-group (pre-post) comparisons and low for between-group comparisons for strength outcomes at ≤ 1 h of recovery, moderate (within) and low (between) for DOMS at 24 h, low to moderate (within) and low (between) for DOMS at 48 h, and low (within and between) for DOMS at 72 h. Information in terms of recovery of ROM after different recovery protocols was insufficient to run a meta-analysis. There was no evidence of publication bias.

### Poor External Validity

Overall, the studies included in our analysis may be considered to have poor external validity. In terms of population, they only apply to adults under 40-years-old, with no studies being performed in children, teenagers or adults older ≥40-years-old. And only two of the 11 studies included women in their sample: 50% of the sample in one study (McGrath et al., 2014) and unclear in another (Bonfim et al., 2010). As such, current results derive mainly from studies with men. As all subjects were healthy, it is unclear how subjects with injuries and/or pathologies would respond. Furthermore, only two studies included recreationally trained subjects (West et al., 2014) or athletes (César et al., 2021).

The nature of the exercise protocols (pre-recovery) presents a number of problems that limit their external validity as well. While most studies used protocols that were likely to induce DOMS, in real-life settings coaches are unlikely to regularly try to elicit DOMS in their athletes or patients. And since most studies did not assess athletes, it is possible that results from the fatigue-inducing protocols have been somewhat artificial, as most were conducted with populations not engaged in regular, structured physical activity, and thereby less well-adapted to the acute effects of fatiguing exercise. Lack of familiarity with the protocols may have exacerbated this effect. Moreover, the protocols were single-component or even single exercise, while real-life exercise sessions will more likely involve multiple components and/or multiple exercises. Also, most of the knowledge derives from studies focusing on the lower limbs, with only one study having assessed the effects of the upper limbs (César et al., 2021).

With one exception (Cooke et al., 2018), the fatigue-inducing protocols had very short durations, usually well below 30 min. Hardly will a real-life exercise session last ≤ 30 min, especially with athletic populations. Conversely the duration of recovery protocols was excessive in many cases, even reaching 30 min in duration (West et al., 2014; Cooke et al., 2018). The combination of very short exercise sessions with long recovery sessions does not seem practical. Also, six studies (~55%) used individualized passive stretching. This means that one supervisor is required for every practitioner, something that will hardly be possible to implement in physical education classes, sports training, and even for the general gym-going population (exceptions would be those with access to a personal trainer).

### Data Is Scarce, Heterogeneous, and Does Not Support Existing Guidelines

Considering that stretching is so often prescribed as a valid protocol for enhancing post-exercise recovery (ACSM, 2018), the reduced number of studies (*n* = 11) and small overall sample (*n* = 289) emerging from our searches, allied with a considerable diversity of exercise and post-exercise recovery protocols, demonstrate that data is too scarce and heterogeneous to support existing guidelines. Although absence of evidence is not evidence of absence, world-leading organizations should encourage further research in this field before promoting more definitive recommendations. Recommendations should not be provided in the absence of empirical support. At a minimum, guidelines should acknowledge that prescribing post-exercise stretching as a means of improving recovery is based on belief and not on data. In fact, enhancing recovery implies that recovery is accelerated and/or improved if post-exercise stretching is applied than if passive recovery (i.e., rest) is used. Our data does not sustain this belief. Indeed, >70% of the analyzed studies had one group performing passive recovery (i.e., rest), and stretching did not prove to improve recovery when compared to those controls. Perhaps the eventual benefits of post-exercise stretching are balanced by the extra fatigue that they add, although further research is required to better explore the mechanistic phenomena underlying these effects.

We strongly suggest that science should abide by the burden of proof. Until more (and better) data is collected, no case should be built for (or against) post-exercise stretching with the goal of improving recovery. Admittedly, post-exercise stretching may have other goals than improving recovery, but these were not addressed in our analysis.

### What's Different in Relation to Previous Systematic Reviews on the Topic?

As mentioned in the introduction, previous SRMA addressed the topic of post-exercise stretching (Herbert and Gabriel, 2002; Henschke and Lin, 2011; Herbert et al., 2011). However, important differences in design exist in comparison with our review, beyond the natural update: (i) these reviews assessed the effects of both post- and pre-exercise stretching, while we focused solely on post-exercise stretching; (ii) they assessed the effects of stretching on DOMS and risk of injury, while we focused on DOMS, strength levels, and ROM; (iii) finally, they accepted non-randomized studies, while our review was limited to randomized studies; (iv) furthermore, we consulted more databases than those reviews. Therefore, it is not surprising that the list of included articles is largely different. Still, our review reinforces previous conclusions that post-exercise stretching does not confer protection from DOMS, while also showing that it does not accelerate (nor impairs) recovery and strength levels or ROM.

## Limitations

The limited number of studies; the high RoB and high heterogeneity, allied to the diversity of designs and poor external validity advise against more definitive conclusions. Moreover, the included studies solicited extremely varied stretching intensities, but all were based in vague sentences to suggest the subjects the degree of stretching intended. And if stretching intensity is not properly described, any comparisons can be limited (Sands et al., 2013). Instead, we believe that stretching intensity could be more rigorously assessed with instruments such as the Stretching Intensity Scale (Freitas et al., 2015).

## Conclusions

Overall, our data does not support nor contradicts the utilization of post-exercise stretching. Notwithstanding, if post-exercise stretching does not seem to enhance recovery in relation to passive recovery (i.e., rest), the implementation of the former among participants or athletes is, at least, questionable. Still, data is scarce, heterogenous, and overall confidence in cumulative evidence is very low. For now, recommendations on whether post-exercise stretching should be applied for the purposes of recovery are misleading, as the (insufficient) data that is available does not support those claims.

We suggest that future research on post-exercise recovery always pre-registers the protocol and adopts a randomized design, with proper description of how randomization was performed and whether allocation sequence was concealed. A passive recovery (i.e., rest) control group should always be included. Multi-component exercise sessions lasting ≥60 min, with recovery protocols lasting ≤ 15 min, would provide greater external validity to the findings. Studies with women and athletes should be reinforced, as studies with children, teenagers, adults ≥40 years and populations with pathologies and/or injuries are lacking and should be prioritized.

## Data Availability Statement

The original contributions presented in the study are included in the article/Supplementary Materials, further inquiries can be directed to the corresponding author.

## Author Contributions

All authors had substantial contributions to the conception or design of the work, acquisition, analysis, or interpretation of data for the work, drafting the work or revising it critically for important intellectual content, final approval of the version to be published, and agreement to be accountable for all aspects of the work in ensuring that questions related to the accuracy or integrity of any part of the work are appropriately investigated and resolved.

## Conflict of Interest

The authors declare that the research was conducted in the absence of any commercial or financial relationships that could be construed as a potential conflict of interest.
