# Supplementary material for: The Effectiveness of Post-exercise Stretching in Short-Term and Delayed Recovery of Strength, Range of Motion and Delayed Onset Muscle Soreness: A Systematic Review and Meta-Analysis of Randomized Controlled Trials
Source: Front Physiol. 2021 May 5;12:677581. doi: 10.3389/fphys.2021.677581 (PMC8133317; doi:10.3389/fphys.2021.677581)
Supplement: Supplementary file 1 [file Data_Sheet_1.docx]

**Supplementary material**

***Information sources – specificities for the different databases***

Specificities for the different databases: (i) in EBSCO and Scielo, title and abstract had to be searched separately, and so different combinations were required; (ii) in PEDro and PubMed, search was done selecting title/abstract, not keywords; (iii) in Web of Science the combination of title, abstract and keywords was termed “topic”. No filters were applied in any database. Following existing guidelines, a wide net was cast (Higgins et al., 2019). In line with this philosophy, there were two post-protocol registration changes. First, in Scielo all eight planned combinations retrieved zero results. Therefore, we decided to remove some constraints: (i) search in Scielo was not limited to title or abstract, and instead accepted all indexes; (ii) we removed the third search field (“random*”). This allowed the search in Scielo to return results that could be included in the screening process.

In PEDro, the search engine did not recognize some terms and combinations. Again, we decided to use a less narrow search: (i) the second and third fields of search were ignored, and the records only had to include terms from the first field *(“stretch*” OR “flex*” OR “mobility” OR “range of motion”)*; (ii) because PEDro does not allow using Boolean operators directly in the search, these terms were introduced as *stretch* flex* mobility “range of motion”* and the engine was programmed to “match any search term (OR)”. In the February 16, 2021 updated, PEDRO retrieved found 270 records when date of entry was limited to December 25, 2020; however, when date limits were removed, PEDro assumed only the search terms and delivered the same 21 results as in the initial search.

***Search strategy – specific example as required by PRISMA guidelines***

Search strategy for PubMed on December 24, 2020: *(("stretch*"[Title/Abstract] OR "flex*"[Title/Abstract] OR "mobility"[Title/Abstract] OR "range of motion"[Title/Abstract]) AND ("post-exerci*"[Title/Abstract] OR "post-workout"[Title/Abstract] OR "post-exertion"[Title/Abstract] OR "post-train*"[Title/Abstract] OR "after exerci*"[Title/Abstract] OR "after workout"[Title/Abstract] OR "after exertion"[Title/Abstract] OR "after training"[Title/Abstract] OR "recover*"[Title/Abstract] OR "warm-down"[Title/Abstract] OR "cool-down"[Title/Abstract])) AND ("random*"[Title/Abstract])*. No filters were applied, and 2421 results were provided.

***Planned moderator analyses***

Using a random-effects model and independent computed single factor analysis, potential sources of heterogeneity likely to influence the effects of interventions were selected *a priori*.

*Subgroup analyses*

As the post-exercise recovery responses may be affected by participants age, training status (e.g., athletes vs. sedentary), health status and sex, these factors were considered as potential moderator variables. Sub-group analyses were also planned according to the studies’ RoB as assessed through RoB 2 (Sterne et al., 2019), as well as RCT design, i.e., parallel versus cross-over (Elbourne et al., 2002).

*Single intervention factor analysis*

Single intervention factor analyses were computed for stretching modality (i.e., passive, static, dynamic, PNF), comparator modality (e.g., massage, passive rest, low-intensity cycling), training modality (e.g., endurance, balance, sports) preceding stretching, and duration and intensity of exercise preceding stretching, based on the reported influence of these variables on post-exercise recovery responses (Van Hooren & Peake, 2018).

When appropriate, subgroup analyses and single training factor analyses were divided using the median split technique (Moran, Clark, Ramirez-Campillo, Davies, & Drury, 2019; Moran et al., 2018; Moran et al., 2017). The median was calculated if at least three studies provided data for a given moderator. Of note, when two experimental groups (with the same information for a given moderator) were included in a study, only one of the groups was considered to avoid an augmented influence of the study on the median calculation. In addition, to minimize heterogeneity, instead of using a global median value for a given moderator (e.g., median age, derived from all included studies), median values were calculated considering only those studies that provided data for the outcome being analysed.

*Meta-regression*

A multivariate random-effects meta-regression was conducted to verify if any of the intervention variables (e.g., stretching modality, comparator modality, and training modality preceding stretching) predicted the effects of post-exercise stretching on outcome measures. Computation of meta-regression was performed with at least 10 studies per covariate (Higgins et al., 2019).

***Supplementary Table 1***

Reasons for excluding papers in the screening stage (analysis of title and abstract)

| **Rule** | **Exclusion criteria** | **Excluded records (*n*)^§^** |
| --- | --- | --- |
| **Study type** | Book, book section/chapter, book review.  Commentary, editorial, opinion, letter, note, short survey.  Conference paper, abstracts published in journals.  Errata, corrigendum.  Feasibility and/or pilot studies.*  Guidelines, policy statements, reports.  Magazine articles.  Patent registration.  Retraction or withdrawal (plus original study).  Reviews (any kind), meta-analysis.  Study design, study protocol, trial registration.  Thesis.  Other unpublished work. | 206  72  338  4  83  7  4  144  11  673 + 8  304 + 6  41  25 |
| **Out of scope** | Non-exercise related studies.  Exercise-related studies that were out of scope (e.g., longitudinal effects of training programs, perioperative training programs; themes not related to our topic). | 3978 + 60  1503 + 40 |
| **Participants** | Non-human animals (e.g., rats). | 1 |
| **Interventions** | No post-exercise stretching intervention, e.g.:   - Stretching as the training intervention *per se*. - Stretching applied pre-exercise (e.g., warm-up). - Multimodal interventions (e.g., stretching combined with massage). - Stretching applied multiple times per day before the relevant timepoints (e.g., 24h). - No stretching intervention at all (most of the studies in this category). | 274 + 5 |
| **Comparators** | Absence of comparators (i.e., single-group observational studies).  Multimodal comparators that also include stretching. | 1 |
| **Outcomes** | No outcomes related to strength and/or ROM for short-term recovery.  AND  no outcomes related to DOMS, strength and/or ROM for delayed recovery. | 2 + 1 |
| **Study design** | Non-randomized studies.  Non-supervised intervention and/or comparators.  Case reports, case series, observational studies (e.g., case-control and cohort studies). | — |
| **Timeframe for follow-up** | No study will be excluded if presenting values >72h, but these will not be considered for analysis. | — |
| **Untraceable full text** | The full texts could not be found, even in the journal websites. Links were disable and, in some cases, the studies did not even appear in Google searches or in new searches in the databases from where they had been originally exported. | 2 |

*We verified if feasibility and/or pilot studies were within the scope of our PICOS criteria, which they were not.

§ Most numbers refer to the first round of searches. Whenever a ‘+’ sign appears, it means that the second number refers to the updated searches.

***Supplementary table 2***

Risk of bias in individual studies (one assessment per outcome).

| **Study** | **D1** | **D2** | **D3** | **D4** | **D5** | **DS** | **Overall** |
| --- | --- | --- | --- | --- | --- | --- | --- |
| Bonfim et al. (2010)  *Pain (pressure dorimeter)* |  |  |  |  |  | N/A |  |
| Bonfim et al. (2010)  *Pain (VAS)* |  |  |  |  |  | N/A |  |
| Cè et al. (2013)  *Maximum voluntary contraction* |  |  |  |  |  |  |  |
| Cè et al. (2013)  *Stand and reach* |  |  |  |  |  |  |  |
| César et al. (2021)  *Handgrip-based measures* |  |  |  |  |  | N/A |  |
| Cooke, Nix, Greenwood, and Greenwood (2018)  *Perceived muscle soreness* |  |  |  |  |  | N/A |  |
| Cooke et al. (2018)  *Isokinetic torque testing* |  |  |  |  |  | N/A |  |
| Kokkinidis, Tsamourtas, Buckenmeyer, and Machairidou (1998)  *Strength (leg curl)* |  |  |  |  |  | N/A |  |
| Kokkinidis et al. (1998)  *Sit and reach* |  |  |  |  |  | N/A |  |
| Kokkinidis et al. (1998)  *Pain questionnaire* |  |  |  |  |  | N/A |  |
| McGrath, Whitehead, and Caine (2014)  *Sit and reach* |  |  |  |  |  | N/A |  |
| McGrath et al. (2014)  *Muscle soreness scale* |  |  |  |  |  | N/A |  |
| Mika, Mika, Fernhall, and Unnithan (2007)  *Maximum voluntary contraction* |  |  |  |  |  |  |  |
| Muanjai and Namsawang (2015)  *Soreness (VAS)* |  |  |  |  |  | N/A |  |
| Muanjai and Namsawang (2015)  *Knee flexion – range of motion* |  |  |  |  |  | N/A |  |
| Muanjai and Namsawang (2015)  *Maximal isometric contraction* |  |  |  |  |  | N/A |  |
| Muanjai and Namsawang (2015)  *Vertical jump* |  |  |  |  |  | N/A |  |
| Torres, Carvalho, and Duarte (2005)  *Pain (VAS)* |  |  |  |  |  | N/A |  |
| Torres et al. (2005)  *Isokinetic peak torque* |  |  |  |  |  | N/A |  |
| Torres, Pinho, Duarte, and Cabri (2013)  *Maximal concentric peak torque* |  |  |  |  |  | N/A |  |
| Torres et al. (2013)  *Soreness (VAS)* |  |  |  |  |  | N/A |  |
| West, Cooke, LaBounty, Byars, and Greenwood (2014)  *Power output-related variables in cycle ergometer* |  |  |  |  |  |  |  |

D1 – Randomization process. D2 – Deviations from intended intervention – effect of assignment to intervention. D3 – Missing outcome data. D4 – Measurement of the outcome. D5 – Selection of the reported result. DS – Domain S – Bias arising from period and crossover effects; specific to crossover designs and not applicable to parallel trials. VAS – Visual analogue scale. N/A – Not applicable. Colors: green means low risk of bias; yellow means some concerns; red means high risk of bias.

**References**

Bonfim, A. E. D., De Re, D., Gaffuri, J., Costa, M. M. D., Portolez, J. L. M., & Bertolini, G. R. F. (2010). Use of Static Stretching as an Intervenient Factor in Delayed Onset Muscle Soreness. *Revista Brasileira De Medicina Do Esporte, 16*(5), 349-352. Retrieved from <Go to ISI>://WOS:000283858600006

Cè, E., Limonta, E., Maggioni, M. A., Rampichini, S., Veicsteinas, A., & Esposito, F. (2013). Stretching and deep and superficial massage do not influence blood lactate levels after heavy-intensity cycle exercise. *Journal of Sports Sciences, 31*(8), 856-866. doi:10.1080/02640414.2012.753158

Cooke, M. B., Nix, C. M., Greenwood, L. D., & Greenwood, M. C. (2018). No differences between alter G-trainer and active and passive recovery strategies on isokinetic strength, systemic oxidative stress and perceived muscle soreness after exercise-induced muscle damage. *Journal of strength and conditioning research, 32*(3), 736-747. doi:10.1519/jsc.0000000000001750

Elbourne, D. R., Altman, D. G., Higgins, J. P. T., Curtin, F., Worthington, H. V., & Vail, A. (2002). Meta-analyses involving cross-over trials: methodological issues. *International Journal of Epidemiology, 31*(1), 140-149. doi:10.1093/ije/31.1.140

Higgins, J. P., Thomas, J., Chandler, J., Cumpston, M., Li, T., Page, M. J., & Welch, V. (2019). *Cochrane Handbook for Systematic Reviews of Interventions (2nd Ed.)*. Chichester (UK): John Wiley & Sons.

Kokkinidis, E., Tsamourtas, A., Buckenmeyer, P., & Machairidou, M. (1998). The effect of static stretching and cryotherapy on the recovery of delayed muscle soreness. *Exercise & Society Journal of Sport Science*(19), 45-53.

McGrath, R. P., Whitehead, J. R., & Caine, D. J. (2014). The Effects of Proprioceptive Neuromuscular Facilitation Stretching on Post-Exercise Delayed Onset Muscle Soreness in Young Adults. *International Journal of Exercise Science, 7*(1), 14-21.

Mika, A., Mika, P., Fernhall, B., & Unnithan, V. B. (2007). Comparison of recovery strategies on muscle performance after fatiguing exercise. *Am J Phys Med Rehabil, 86*(6), 474-481. doi:10.1097/PHM.0b013e31805b7c79

Moran, J., Clark, C. C. T., Ramirez-Campillo, R., Davies, M. J., & Drury, B. (2019). A Meta-Analysis of Plyometric Training in Female Youth: Its Efficacy and Shortcomings in the Literature. *J Strength Cond Res, 33*(7), 1996-2008. doi:10.1519/jsc.0000000000002768

Moran, J., Sandercock, G., Ramirez-Campillo, R., Clark, C. C. T., Fernandes, J. F. T., & Drury, B. (2018). A Meta-Analysis of Resistance Training in Female Youth: Its Effect on Muscular Strength, and Shortcomings in the Literature. *Sports Medicine, 48*(7), 1661-1671. doi:10.1007/s40279-018-0914-4

Moran, J., Sandercock, G. R., Ramírez-Campillo, R., Meylan, C., Collison, J., & Parry, D. A. (2017). A meta-analysis of maturation-related variation in adolescent boy athletes' adaptations to short-term resistance training. *J Sports Sci, 35*(11), 1041-1051. doi:10.1080/02640414.2016.1209306

Muanjai, P., & Namsawang, J. (2015). Effects of stretching and cold-water immersion on functional signs of muscle soreness following plyometric training. *Journal of Physical Education and Sport, 15*(1), 128-135. doi:10.7752/jpes.2015.01021

Sterne, J. A. C., Savović, J., Page, M. J., Elbers, R. G., Blencowe, N. S., Boutron, I., . . . Higgins, J. P. T. (2019). RoB 2: a revised tool for assessing risk of bias in randomised trials. *BMJ, 366*, l4898. doi:10.1136/bmj.l4898

Torres, R., Carvalho, P., & Duarte, J. A. (2005). Effects of a static stretching program on clinical and biochemical markers of muscle damage induced by eccentric exercise. *Revista Portuguesa de Ciências do Desporto, 5*, 274-287.

Torres, R., Pinho, F., Duarte, J. A., & Cabri, J. M. H. (2013). Effect of single bout versus repeated bouts of stretching on muscle recovery following eccentric exercise. *Journal of Science and Medicine in Sport, 16*(6), 583-588. doi:10.1016/j.jsams.2013.01.002

Van Hooren, B., & Peake, J. M. (2018). Do We Need a Cool-Down After Exercise? A Narrative Review of the Psychophysiological Effects and the Effects on Performance, Injuries and the Long-Term Adaptive Response. *Sports Med, 48*(7), 1575-1595. doi:10.1007/s40279-018-0916-2

West, A. D., Cooke, M. B., LaBounty, P. M., Byars, A. G., & Greenwood, M. (2014). Effects of G-trainer, cycle ergometry, and stretching on physiological and psychological recovery from endurance exercise. *J Strength Cond Res, 28*(12), 3453-3461. doi:10.1519/jsc.0000000000000577
